# Programmed Death-Ligand 1 (PD-L1) Expression Is Induced by Insulin in Pancreatic Ductal Adenocarcinoma Cells Pointing to Its Role in Immune Checkpoint Control

**DOI:** 10.3390/medsci9030048

**Published:** 2021-06-25

**Authors:** Steffen M. Heckl, Franziska Mau, Anke Senftleben, Tina Daunke, Silje Beckinger, Samir Abdullazade, Stefan Schreiber, Christoph Röcken, Susanne Sebens, Heiner Schäfer

**Affiliations:** 1 Department of Internal Medicine I, UKSH Campus Kiel, Arnold-Heller-Str. 3, Bldg. K3, 24105 Kiel, Germany; steffen.heckl@uksh.de (S.M.H.); stefan.schreiber@uksh.de (S.S.); 2 Department of Internal Medicine II, UKSH Campus Kiel, university, Arnold-Heller-Str. 3, Bldg. E, 24105 Kiel, Germany; 3 Institute of Experimental Cancer Research, UKSH Campus Kiel & Christian-Albrechts-University Kiel, Arnold-Heller-Str. 3, Bldg. U30, 24105 Kiel, Germany; franziska-mau@gmx.de (F.M.); as.senftleben@googlemail.com (A.S.); tina.daunke@email.uni-kiel.de (T.D.); silje.beckinger@email.uni-kiel.de (S.B.); susanne.sebens@email.uni-kiel.de (S.S.); 4 Department of Pathology, Christian-Albrechts-University Kiel, Arnold-Heller-Str. 3, Bldg. U33, 24105 Kiel, Germany; samir.abdullazade@uksh.de (S.A.); christoph.roecken@uksh.de (C.R.)

**Keywords:** T-cells, carcinogenesis, diabetes mellitus, hyperinsulinemia, signal transduction

## Abstract

**Simple Summary:**

Type-2 Diabetes is an established risk factor of pancreatic cancer, yet the exact modalities as well as the contribution of hyperinsulinemia are not fully understood. In our study, we explored a possible link between insulin and the immune evasion of pancreatic cancer cells. We could demonstrate that insulin is able to induce expression of Programmed Death-Ligand 1 (PD-L1) representing a key molecule in immune checkpoint control. This effect of insulin greatly depends on insulin receptor-A and/or IGF-receptor-induced ERK signaling and is enhanced via a crosstalk with EGF. Through insulin-induced PD-L1 expression, pancreatic cancer cells suppress proliferation of CD8+ T-cells underscoring the potential of insulin to confer immune evasion through this immune checkpoint regulator. Furthermore, some cases out of a cohort of pancreatic cancer patients reveal coexpression of PD-L1 and the cytoplasmic insulin receptor in the tumor tissue as shown by immunohistochemistry.

**Abstract:**

Type-2 diabetes (T2DM) is a risk factor for the development of pancreatic ductal adenocarcinoma (PDAC) and is characterized by insulin resistance and hyperinsulinemia. Besides the well-known growth-promoting activity of insulin or the other members of the Insulin/Insulin-like Growth factor (IGF) axis, we here describe an inducing effect of insulin on PD-L1 expression in PDAC cells. Treatment of the PDAC cell lines BxPc3, A818-6, and T3M4 with insulin increased PD-L1 expression in a time- and dose dependent fashion, as shown by Western blot and qPCR analysis. siRNA mediated knock-down showed that the effects of insulin on PD-L1 depend on the insulin and IGF receptors (InsR and IGFR, respectively). In addition, a crosstalk of insulin-induced ERK activation and Epidermal Growth Factor (EGF) triggered PD-L1 expression. This involves different mechanisms in the three cell lines including upregulation of InsR-A expression in A818-6 and modulation of the adaptor protein Gab1 in BxPc3 cells. As a consequence of the insulin-induced PD-L1 expression, PDAC cells suppress the proliferation of activated human CD8+ T-cells in coculture experiments. The suppression of CD8+ cell proliferation by insulin-pretreated PDAC cells was reversed by PD-1 blockade with Pembrolizumab or by PD-L1 siRNA. Furthermore, the clinical relevance of these observations was supported by detecting a coexpression of cytoplasmic InsR (characteristic for its activation) and PD-L1 in tumor tissues from PDAC patients. Our findings provide a novel insight into the protumorigenic role of insulin in PDAC. Recognizing the impact of insulin on PD-L1 expression as part of the immune privilege, strategies to interfere with this mechanism could pave the way towards a more efficient immunotherapy of PDAC.

## 1. Introduction

Pancreatic ductal adenocarcinoma (PDAC) represents the fourth leading cause of cancer-related deaths in Western countries. Due to a difficult and late diagnosis, only 15% of the patients have a disease still localized to the pancreas, allowing a potentially curative resection [1,2,3]. The majority of patients, having a locally advanced tumor status, receive radio- and or chemotherapy [3,4,5]. Gemcitabine or 5-fluoruracil are the most frequently used drugs, but like all other therapeutical interventions they fail to significantly improve the prognosis. Even the recommended first-line mFOLFIRINOX combination therapy (folinic acid, fluorouracil, Irinotecan, oxaliplatin) for patients with a good performance status just improves the life expectancy in patients with locally advanced cancer from 6–7 months seen with gemcitabine therapy to 10–11 months. Despite these recent advances, the overall outcome of the patients remains desperately poor [4,5].

Evidence has accumulated that type 2 diabetes mellitus (T2DM) is one of the risk factors of PDAC [6,7,8,9,10]. Characterized by chronic hyperglycemia, insulin resistance, and hyperinsulinemia, T2DM favors metabolic conditions that considerably affect tumorigenesis. Thus, hyperglycemia was identified to promote pancreatic tumorigenesis [11,12] and metastasis formation—for example, in the liver [13]. Moreover, an altered metabolism from an oxidative to a more glycolytic metabolism was recently associated with a gain in cancer stemness and thereby greater potential in metastasis formation and therapy resistance [14]. Besides the altered glucose metabolism, insulin itself seems to be involved in T2DM-driven pancreatic tumorigenesis [15]. Being part of the so-called insulin–insulin-like growth factor (IGF) axis, insulin and IGF exert growth promoting and antiapoptotic activities in cancer cells that are mediated through a complex signaling network [15,16,17,18]. In addition to the insulin receptors (InsR), receptors for IGF (IGFR) can homodimerize or form heterodimers with InsR giving rise to a complex activation pattern through insulin as well as of IGF1 and IGF2 [16,17]. A particular role is played by the two InsR isoforms InsR-A and InsR-B that structurally differ by just 12 amino acids within the C-terminus of the InsR α-chain and that result from differential mRNA splicing [19,20,21]. The InsR-B mainly drives the metabolic actions of insulin that are greatly mediated through the PI3K/Akt pathway [22,23]. The InsR-A drives mainly the proliferative activity of insulin that involves activation of the Ras/ERK pathway [24]. While the effects of insulin and IGF on tumor cell growth have been recognized already [15,16,17,18], their role in the acquired immune privilege in PDAC is not known yet.

Besides creating an immunosuppressive milieu, for example by the recruitment of regulatory T-cells or inhibitory/type 2 macrophages [25,26], tumor cells engage the immune checkpoint control to evade their elimination by the immune system [27]. This involves the interaction of the surface protein Programmed Death-Ligand 1 (PD-L1) expressed on cancer cells with Programmed Death protein-1 (PD-1) that is co-expressed with CD3/TCR and CD28 by activated T-cells [28]. Through this PD-L1/PD-1 interaction, cancer cells can directly suppress T-cell activity and escape immune control [29,30]. Recent data demonstrated that immune evasion of PDAC may also involve PD-L1 that is expressed on the cancer cells but also in the tumor stroma [31,32]. Through PD-L1, tumor cells not only induce anergy of invading T-cells but also, through reverse signaling, direct modulation of their own phenotype, e.g., stem cell properties [33]. Besides IFNγ, which is a major inducer of PD-L1 [34,35], multiple mechanisms and pathways have been described to control PD-L1 expression [36,37], yet their contribution to the immune checkpoint control in PDAC has not been confirmed [38].

Until now, research on immune checkpoint control in PDAC has not taken its extraordinary local endocrine environment into account. The naturally higher levels of insulin within the pancreatic organ could fuel PDAC progression by promoting immune evasion. In the present study, we therefore tested the hypothesis that insulin contributes to PD-L1-mediated immune checkpoint control in PDAC. We finally show that insulin induces PD-L1 expression in PDAC cells, thereby impairing CD8+ T-cell proliferation. Through InsR-A and IGF receptors, insulin activates the MAPK pathway that impacts PD-L1 expression. From these findings, we could show, for the first time, a novel role of hyperinsulinemia in PDAC, also providing another piece of evidence regarding how T2DM acts as a risk factor of tumor progression in PDAC.

## 2. Results

### 2.1. Insulin Inducible PD-L1 Expression in PDAC Cells

**Figure 1:**
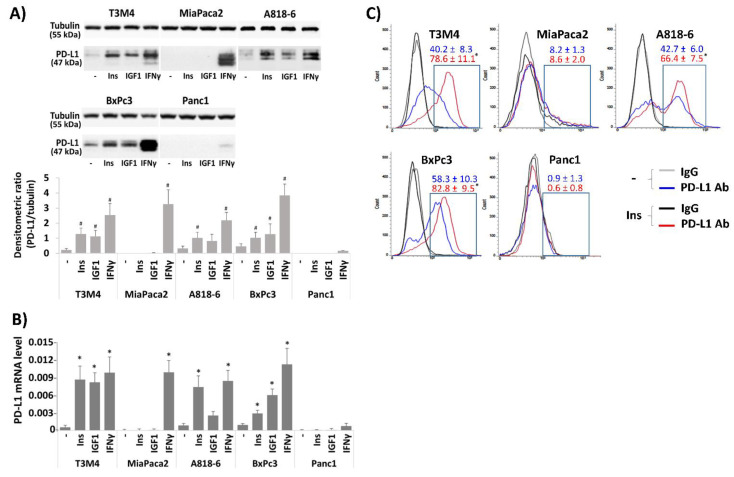
Effect of Insulin and IGF on PD-L1 expression in PDAC cells. (**A**,**B**) the PDAC lines A818-6, T3M4, BxPc3, MiaPaca2, and Panc1 were kept at low FCS concentration (0.3% (*v*/*v*)) for 24 h. Then, cells were either left untreated or were treated for (**A**,**C**) 24 h and (**B**) 16 h with 0.1 IU/mL insulin, 0.1 µg/mL IGF1 or, for comparison, with 50 ng/mL IFNγ. (**A**) Western blot analysis using PD-L1 and Tubulin antibodies. Representative blots from four independent experiments and band densitometry analysis (lower panel, **^#^** *p* < 0.05 compared to untreated) are shown. (**B**) qPCR analysis using PD-L1 and RPL13 primers (the latter used as control). PD-L1 expression was normalized to the expression level of RPL13, and data represent the mean ± SD from four independent experiments (* *p* < 0.03 compared to untreated). (**C**) Non-permeabilized cells were immunostained with anti-PD-L1 or IgG control antibodies and analyzed by fluorescence flow cytometry. A representative result from four independent experiments is shown. The percentage of PD-L1 positive cells (boxed areas) are indicated (mean ± SD, * *p* < 0.05 compared to untreated).

First, we analyzed the basal PD-L1 expression in the PDAC cell lines A818-6, T3M4, and BxPc3 and whether it can be modulated by insulin. In our experiments, we used regular insulin exhibiting maximal activity between 15 min and 3–4 h. Since the presence of serum factors, including IGF, FGF, etc., may interfere with the effect of insulin, all cell lines were cultured with 0.3% (*v*/*v*) FCS for 24 h before administration of insulin. This serum starvation prior to further treatment was applied in all following experiments. As shown by Western blot analysis (Figure 1A), BxPc3 cells showed the highest basal expression of PDL1, followed by A818-6 and T3M4 cells. By contrast, MiaPaca2 and Panc1 cells lacked basal PD-L1 expression. After treatment with 0.1 IU/mL insulin for 24 h, T3M4 cells increased the PD-L1 expression the most, followed by A818-6 cells and BxPc3 cells. In MiaPaca2 and Panc1 cells, no effect of insulin on PDL1 expression was seen. By comparison, treatment with the known PD-L1 inducer IFNγ, acting independently of InsR, led to a strong increase of PD-L1 expression in all cell lines except Panc1 cells (Figure 1A). When we treated these cell lines with 0.1 µL/mL IGF1 as another InsR ligand, PD-L1 expression was elevated in BxPc3 and T3M4 cells to a similar extent as after insulin treatment (Figure 1A). A818-6 cells were less responsive to IGF1 than insulin, whereas both MiaPaca2 and Panc1 cells again showed no induction of PD-L1. Thus, MiaPaca2 cells seem to be refractory to PD-L1 induction by insulin or IGF and Panc1 cells hardly express PD-L1 at all.

The effect of insulin on PD-L1 expression was confirmed by qPCR analysis. As shown in Figure 1B, treatment with insulin at a dose of 0.1 IU/mL for 16 h increased PD-L1 mRNA expression in T3M4, A818-6, and BxPc3 cells 15.1-fold, 8.5-fold, and 3.3-fold, respectively. After IGF1 treatment for the same period, T3M4 cells again revealed the greatest increase of PD-L1 expression (14.4-fold) followed by BxPc3 cells (6.4-fold) and A818-6 cells (3.0-fold induction). Again, MiaPaca2 and Panc1 cells did not respond to insulin or IGF1 treatment. All cell lines, except Panc1 cells, strongly increased PD-L1 mRNA expression after stimulation with IFNγ (Figure 1B). To further characterize the inducing effect of insulin on PD-L1 expression in the three responsive cell lines, its time- and dose dependency was checked by Western blot and qPCR analyses that are shown in the Supplemental Information (Section S2 and Figure S1).

In order to confirm whether insulin treatment also alters PD-L1 surface expression, non-permeabilized PDAC cells were PD-L1 immunostained and analyzed by fluorescence flow cytometry (Figure 1C). When compared with the IgG isotype controls, PD-L1 antibody staining was stronger in A818-6, T3M4, and BxPc3 cells, and insulin treatment for 24 h further increased the PD-L1 surface staining intensity in these cells. By contrast, MiaPaca2 and Panc1 cells did neither show relevant PD-L1 surface staining nor an effect of insulin treatment and were therefore excluded from further experiments.

### 2.2. Differential InsR-A, InsR-B, and IGF-R Expression in PDAC Cells and Their Impact on PD-L1 Expression

**Figure 2:**
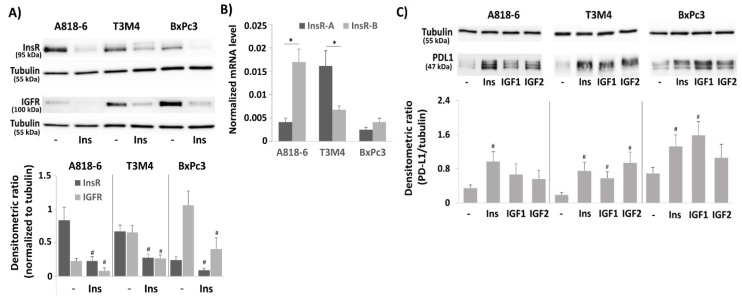

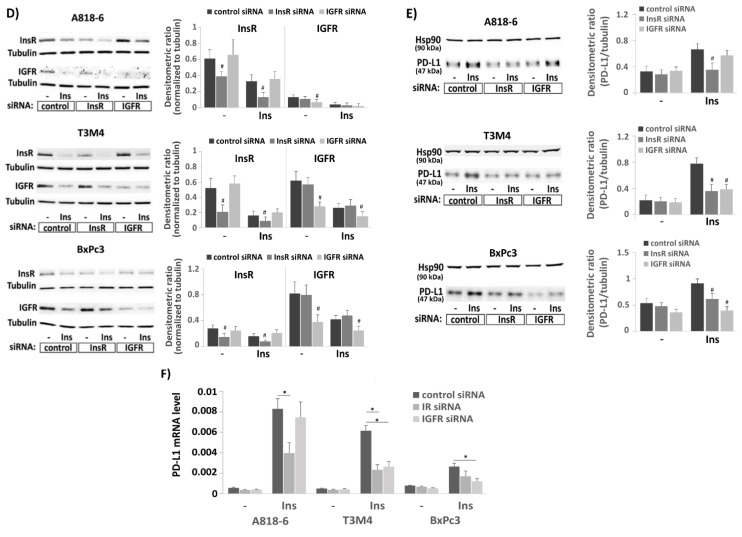
Involvement of insulin receptors (InsR) and IGF receptors (IGFR) in the insulin-induced PD-L1 expression in PDAC cell lines. (**A**–**C**) Serum-starved A818-6, T3M4, and BxPc3 cells were either left untreated (**B**) or were treated (**A**) with 0.1 IU/mL insulin for 24 h and (**C**), with 0.1 IU/mL insulin, 0.1 µg/mL IFG1, or 0.1 µg/mL IGF2 for 24 h. (**A**,**C**) Western blot analysis using (**A**) InsR and tubulin or (**C**) PD-L1 and tubulin antibodies. Representative blots from four independent experiments and band densitometry analyses (lower panels, ^#^ *p* < 0.05 compared to untreated, *n* = 4) are shown. (**B**) qPCR analysis using InsR-A, InsR-B, and RPL13 primers. InsR-A and InsR-B expression was normalized to the expression level of RPL13, and data represent the mean ± SD from five independent experiments (* *p* < 0.03 compared to untreated). (**D**–**F**) A818-6, T3M4, and BxPc3 cells were pretreated with control, InsR, or IGFR siRNA for 48 h, followed by treatment with 0.1 IU/mL insulin or without for 24 h. (**D**,**E**) Western blot analysis using (**D**) InsR, IGFR, and tubulin or (**E**) PD-L1 and Hsp90 antibodies. Representative blots from four independent experiments and band densitometry analyses (right panels, ^#^ *p* < 0.05 compared to control siRNA, *n* = 4) are shown. (**F**) qPCR analysis using PD-L1 and RPL13 primers. PD-L1 expression was normalized to the expression level of RPL13, and data represent the mean ± SD from five independent experiments (* *p* < 0.03 compared to control siRNA).

Next, we analyzed A818-6, T3M4, and BxPc3 cells for the expression of InsR and IGFR. As shown by Western blot (Figure 2A), InsR expression was much higher in A818-6 and T3M4 cells than in BxPc3 cells. IGFR expression was highest in BxPc3 cells followed by T3M4 cells, whereas it was almost absent in A818-6 cells. Typically, treatment with insulin reduced the amount of InsR and IGFR protein as both are internalized and degraded upon ligand binding. Since the two isoforms of the InsR—InsR-A and InsR-B—differ in their signal transduction and could thereby account for the differential responsiveness to insulin in the PDAC cells, qPCR analysis was done to discriminate between these two receptor variants (the antibody used for Western blotting detects both isoforms). As shown in Figure 2B, A818-6 cells expressed InsR-B at a much higher level than InsR-A, whereas T3M4 cells expressed InsR-A at a higher level than InsR-B. By contrast, BxPc3 cells show only low expression of both InsRs. From this expression pattern, it can be assumed that the PD-L1 inducing signal of insulin mainly involves InsR-B homo- and InsR-A/B heterodimers in A818-6, InsR-A/IGFR heterodimers in T3M4, and IGFRs in BxPc3 cells. This was also supported by the observation that in InsR-A-expressing T3M4 cells, IGF2 showed a greater effect on PD-L1 expression than IGF1, whereas the effect of IGF2 was weaker than IGF1 in A818-6 cells and BxPc3 cells (Figure 2C).

To validate the PD-L1-inducing effect of insulin and to analyze which receptors are involved in it, InsR or IGFR were knocked-down by siRNA (Figure 2D). As can be appreciated from Figure 2E,F, in all three cell lines, InsR knock-down diminished insulin-induced PD-L1 expression. Both protein and mRNA level of PD-L1 were less elevated 24 h after insulin administration (0.1 IU/mL) when the cells were pretreated with InsR siRNA as compared to cells pretreated with the control siRNA. By contrast, in A818-6 cells, the effect of insulin on PD-L1 expression was not efficiently blocked by the IGFR knock-down, which is in line with their low IGFR expression (Figure 2A,B). In T3M4 cells, the effect of InsR and IGFR knock-down was similar, whereas in BxPc3 cells, the knock-down of IGFR (Figure 2D) had a stronger effect on insulin-induced PD-L1 expression (Figure 2E,F) than the InsR knock-down.

### 2.3. Signal Transduction Underlying the Effect of Insulin on PD-L1 Expression in PDAC Cells

**Figure 3:**
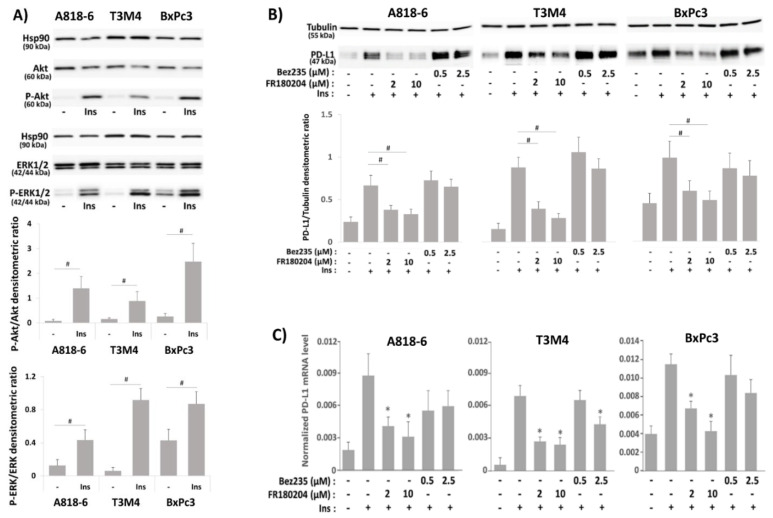
Signal transduction underlying the effect of insulin on PD-L1 expression in PDAC cells. (**A**) Serum-starved A818-6, T3M4, and BxPc3 cells were either left untreated or were treated for 20 min with 0.1 IU/mL insulin. (**B**,**C**), Serum-starved A818-6, T3M4, and BxPc3 cells either pretreated with the ERK1/2 inhibitor FR180204 (2 µM or 10 µM) or the PI3K inhibitor Bez235 (0.5 µM or 2.5 µM) for 1 h, or left without pretreatment were treated for (**B**) 24h or (**C**) 16 h with 0.1 IU/mL insulin or left without any treatment. (**A**,**B**) Western blot analysis using (**A**) anti P-ERK1/2, P-Akt, ERK1/2, Akt, and Hsp90 antibodies or (**B**) PD-L1 and tubulin antibodies. Representative blots from four independent experiments and band densitometry analyses (lower panels, **^#^** *p* < 0.05, *n* = 4) are shown. (**C**) qPCR analysis using PD-L1 and RPL13 primers was performed. PD-L1 expression was normalized to the expression level of RPL13, and data represent the mean ± SD from four independent experiments (* *p* < 0.03 compared to insulin treatment alone).

We next explored the involvement of the PI3K/Akt and MAPK/ERK signaling pathways in the PD-L1 induction by insulin. As shown in Figure 3A, phospho-Akt expression was strongly induced after insulin treatment (0.1 IU/mL for 30 min) in A818-6 and BxPc3 cells, but to a lesser extent in T3M4 cells. By contrast, phospho-ERK1/2 expression was strongly induced by insulin in T3M4 cells (Figure 3A), whereas a weaker induction was seen in A818-6 and BxPc3 cells, which already exhibited higher basal phospho-ERK1/2 level. As further shown by Western blot and qPCR analysis (Figure 3B,C), the PI3K inhibitor Bez325 or the ERK1/2 inhibitor FR180204 (efficacy testing is shown in Figure S2 in the Supplemental Information) differentially affected the insulin-mediated induction of PD-L1 expression when added 1 h before insulin administration. While Bez325 did not alter or even increased the effect of insulin treatment on PD-L1 expression in the analyzed PDAC cell lines, FR180204 preincubation abolished the induction of PD-L1 expression by insulin in T3M4, A818-6, and BxPc3 cells in a dose-dependent fashion.

### 2.4. Crosstalk of Insulin and EGF in Inducing PD-L1 Expression in PDAC Cells

**Figure 4:**
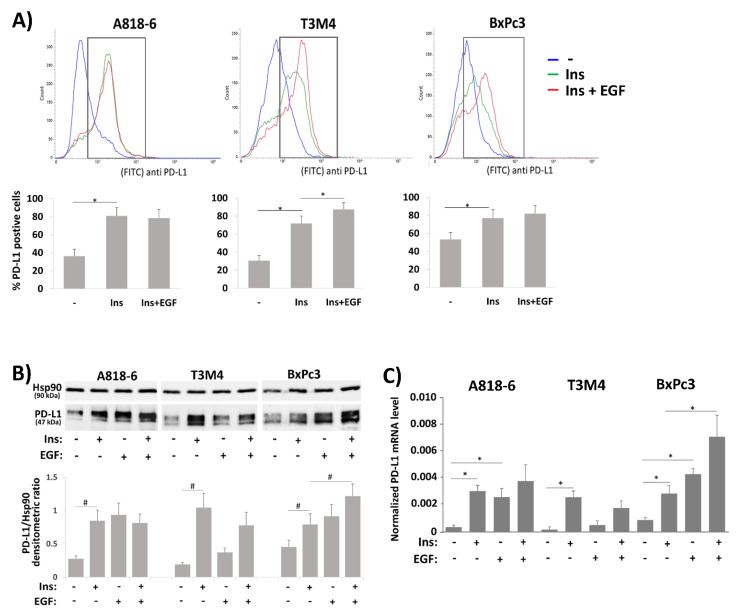

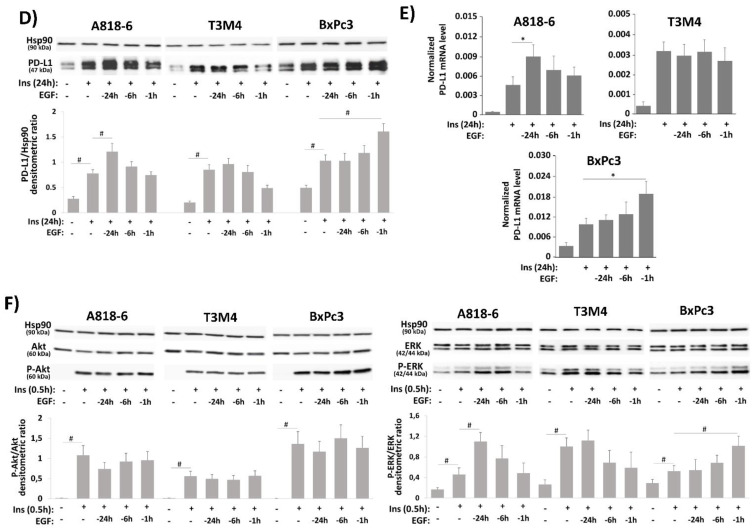
Crosstalk of insulin and EGF in inducing PD-L1 expression in PDAC cells. (**A**–**C**) Serum-starved A818-6, T3M4, and BxPc3 cells were either left untreated or treated with 0.1 IU/mL insulin for 24 h (**A**,**B**) or 16 h (**C**) either alone or in the presence of 10 ng/mL EGF. (**A**) Immunostaining of surface PD-L1 expression and fluorescence flow cytometry. A representative result from five independent experiments is shown. The percentage of PD-L1 positive cells (boxed areas in the histograms) are depicted in the graph below (mean ± SD, *n* = 5, * *p* < 0.05). (**B**) Western blot analysis using PD-L1 and Hsp90 antibodies. (**C**) qPCR analysis using PD-L1 and RPL13 primers. (**D**,**E**) Serum-starved A818-6, T3M4, and BxPc3 cells were insulin treated as above either alone or following preincubation with 10 ng/mL EGF for the indicated periods. Then, cells were submitted to (**D**) Western blot analysis using PD-L1 and Hsp90 antibodies or (**E**) to qPCR analysis using PD-L1 and RPL13 primers. (**F**) Serum-starved A818-6, T3M4, and BxPc3 cells were either left untreated or were treated with 0.1 IU/mL insulin for 20 min either alone or after preincubation with 10 ng/mL EGF for the indicated periods. Then, Western blot analysis for P-ERK1/2, ERK1/2, P-Akt, Akt, and Hsp90 was performed. (**B**,**D**,**F**) Representative blots from four independent experiments and band densitometry analyses (lower panels, **^#^** *p* < 0.05, *n* = 4) are shown. (**C**,**E**) PD-L1 mRNA expression is normalized to the expression level of RPL13, and data represent the mean ± SD from four independent experiments; (* *p* < 0.02).

Recently, it was reported that insulin and EGF synergize in activating PD-L1 expression in colon cancer cells, an effect that involves the enhanced transport of PD-L1 to the cell surface [29]. We therefore elucidated whether concomitant EGF treatment or pretreatment with EGF alters the inducing effect of insulin on PD-L1 in the three PDAC cell lines. At first, fluorescence flow cytometry analysis of unpermeabilized and anti-PDL1 immunostained cells was carried out (Figure 4A). In A818-6, T3M4, and BxPc3 cells, insulin treatment led to an increase of PD-L1 surface expression that was enhanced by EGF co-treatment in T3M4 and BxPc3 cells. In A818-6 cells, no increasing effect by EGF on insulin-induced PD-L1 surface expression was seen.

As shown by Western blot and qPCR (Figure 4B,C), administration of EGF alone increased PD-L1 expression in A818-6 to a similar extent as insulin but the combination of both revealed no stronger effect on PD-L1 expression. In BxPc3 cells, EGF more efficiently induced PD-L1 expression than insulin and the combination of both greatly enhanced it. By contrast, PD-L1 expression in T3M4 cells was only moderately induced by EGF treatment compared to the much stronger effect by insulin treatment, and the combined treatment with insulin and EGF resulted in a somewhat weaker PD-L1 induction.

Next, EGF treatment was conducted at different time points before insulin administration. As shown in Figure 4D,E, PD-L1 expression in A818-6 cells was most strongly enhanced by longer EGF pretreatment (-24 h and −16 h respectively), whereas in BxPc3 cells, only the shorter preincubation (−1 h) enhanced the insulin-induced PD-L1 expression. In T3M4 cells, the insulin-induced PD-L1 expression became even weaker when EGF was administered 1 h before. Regarding the phospho-ERK1/2 expression level (Figure 4F), the inducing effect of insulin was enhanced by long-term pretreatment with EGF (−24 h and −6 h) in A818-6 cells whereas in BxPc3 cells, particularly the short-term pretreatment (−1 h) with EGF led to a strong increase of the insulin-induced phospho-ERK1/2 level. In T3M4 cells, EGF pretreatment (−6 h and −1 h) even reduced the insulin-induced phospho-ERK1/2. Under the same conditions, the level of insulin-induced phospho-Akt was only moderately affected by EGF pretreatment.

### 2.5. The Effect of EGF on Insulin-Induced PD-L1 Expression in PDAC Cells Involves Different Mechanisms

**Figure 5:**
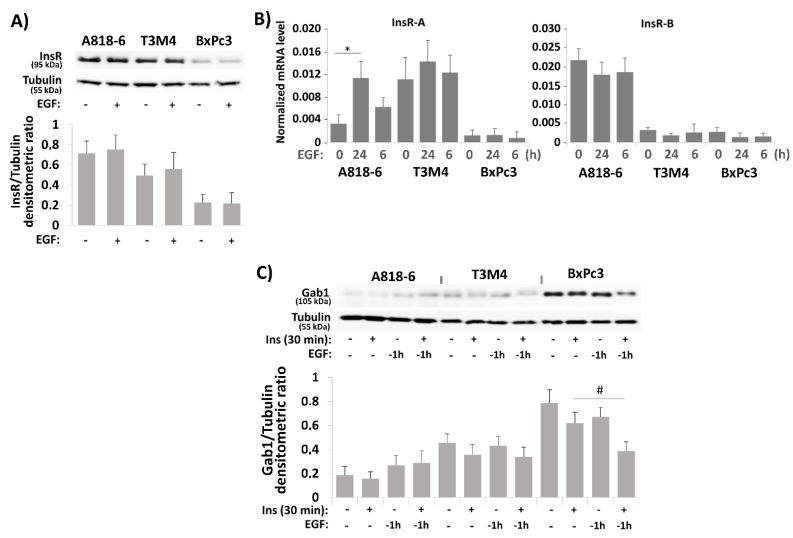

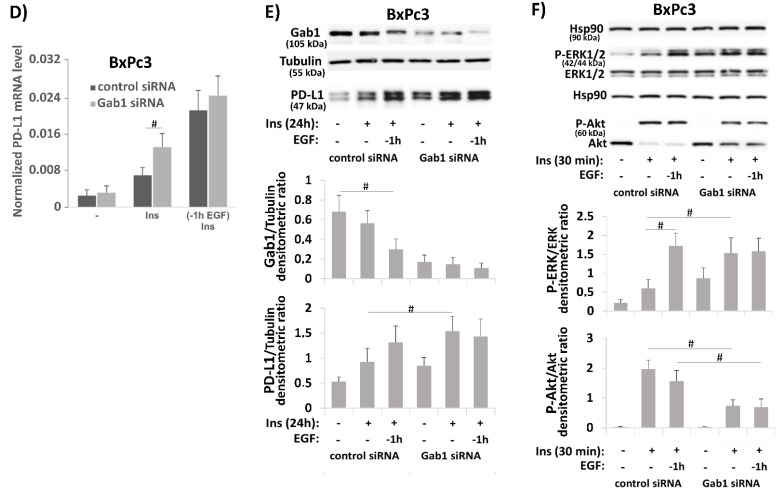
The effect of EGF on insulin-induced PD-L1 expression in PDAC cells involves different mechanisms. (**A**,**B**) Serum-starved A818-6, T3M4, and BxPc3 cells were either left untreated or treated with 10 ng/mL EGF for (**A**) 24 h or (**B**) 6 h and 24 h. (**A**) Western blot analysis using InsR and tubulin antibodies. (**B**) qPCR analysis using InsR-A, InsR-B, and RPL13 primers. InsR-A and InsR-B expression is normalized to the expression level of RPL13, and data represent the mean ± SD from four independent experiments (* *p* < 0.02 compared to untreated). (**C**) Serum-starved A818-6, T3M4, and BxPc3 cells were either left untreated or treated with 0.1 IU/mL insulin for 30 min either alone or after preincubation for the indicated periods with 10 ng/mL EGF. Then, Western blot analysis using Gab1 and tubulin antibodies was performed. (**D**–**F**) BxPc3 cells were treated with control or Gab1 siRNA for 24 h followed by serum starvation for 24 h. Then, cells either left untreated or were treatment with 0.1 IU/mL insulin for 16 h (**D**), 24 h (**E**) or 30 min (**F**) either in the absence or presence of 10 ng/mL EGF administered 1 h before. (**D**) qPCR analysis using PD-L1 and RPL13 primers. PD-L1 expression is normalized to the expression level of RPL13, and data represent the mean ± SD from four independent experiments (* *p* < 0.05). (**E**,**F**) Western blot analysis using (**E**) PD-L1, Gab1, and tubulin antibodies or (**F**) P-ERK1/2, ERK1/2, P-Akt, Akt, and Hsp90 antibodies. (**A**,**C**,**E**,**F**) Representative blots from four independent experiments and band densitometry analyses (lower panels, **^#^** *p* < 0.05, *n* = 4) are shown.

In contrast to T3M4 cells that reveal an enhancing effect of EGF on insulin-induced PD-L1 surface expression but not its general expression level (see Figure 4), A818-6 and BxPc3 cells clearly showed an EGF effect on the insulin-induced PD-L1 expression level that is largely dispensable from its transport to the cell surface [33] but exhibits a different time dependency. A possible mechanism by which EGF potentiates the increasing effect of insulin on PD-L1 expression could be its inducing impact on the expression of InsR-A [39]. As shown in Figure 5A, Western blot analysis revealed no substantial differences in InsR expression in the three cell lines when treated with EGF. By contrast, qPCR analysis unraveled an increase of InsR-A and a decrease of InsR-B expression in A818-6 cells, an effect also seen to a lesser extent in T3M4 cells but not in BxPc3 cells (Figure 5B). Thus, the differential InsR-A expression in response to EGF could be responsible for the enhanced effect of insulin on PD-L1 expression, particularly in A818-6 cells, whereas in T3M4 cells that express already higher basal level of InsR-A, EGF was not effective through this mechanism. In BxPc3 cells, however, another mechanism seems to be involved.

In a previous study, it was shown that the crosstalk between EGF and insulin involves the Grb2 associated binder-1 (Gab1) protein that upon EGFR activation is recruited to the InsR-B, thereby leading to a shift of the insulin driven signaling from the PI3K- towards the ERK-pathway [40]. Therefore, we analyzed the Gab1 expression after administration of insulin or EGF alone for 0.5 and 1 h, respectively, as well as insulin following 1 h preincubation with EGF. As could be appreciated from Western blot (Figure 5C), in A818-6 and T3M4 cells, only a faint basal Gab1 expression was detectable that was slightly affected by EGF or combined EGF/insulin treatment. In striking contrast, BxPc3 cells revealed a much stronger basal expression of Gab1. Here, EGF treatment altered Gab 1 expression giving rise to a Gab1 protein band at slightly higher molecular weight (indicating its phosphorylation and activation [41]) along with a decline of the Gab1 protein amount (indicating forced proteasomal degradation of the activated protein [42]). Moreover, when combined with insulin, the EGF effect on the Gab1 protein was greatly enhanced (Figure 5C). By contrast, mRNA level of Gab1 neither differed considerably in the three cell lines nor did EGF or insulin alter Gab1 mRNA expression (data not shown). This indicates that Gab1 is tightly regulated on protein level, which differentially manifests in the PDAC cells. Interestingly, the knock-down of Gab1 by siRNA enhanced the insulin inducing effect on the PD-L1 expression in BxPc3 cells as shown by qPCR and Western blot analysis (Figure 5D,E). In addition, stronger phospho-ERK1/2 and weaker phospho-Akt expression was seen after Gab1 knock-down (Figure 5F). Thus, in BxPc3 cells, the insulin-induced PD-L1 expression is enhanced by EGF pretreatment through interference with the Gab1-dependent control of insulin-induced signaling. Resulting from that, insulin signaling involves stronger phospho-ERK1/2 activation.

### 2.6. Transcriptional Control of PD-L1 Expression by Insulin Depends on an Enhancer Element in the PD-L1 Promoter

**Figure 6:**
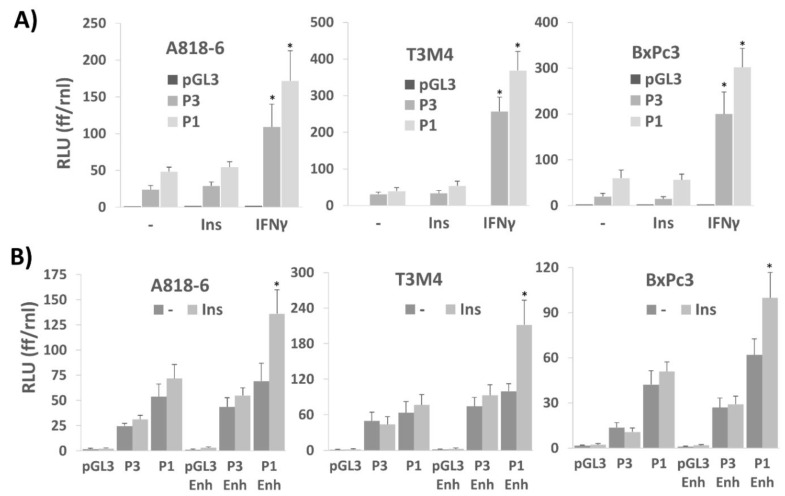
Transcriptional control of PD-L1 expression by insulin depends on an enhancer element in the PD-L1 promoter. A818-6, T3M4, and BxPc3 cells were transfected with (**A**) the 1.3 kb (P1) or the 0.5 kb (P3) PD-L1 promoter constructs driving firefly luciferase or with the empty vector (pGL3), or (**B**) with the 1.3 kb (P1), 0.5 kb (P3) PD-L1 promoter constructs or the empty vector (pGL3) either without or with the 0.23 kb 3′enhancer element (P1 Enh, P3 Enh, and pGL3 Enh, respectively) or the empty vector (pGL3) without or with the enhancer. After 16 h, cells were serum starved for 24 h followed by treatment with 0.1 IU/mL insulin (**A**,**B**) 50 ng/mL IFNγ (**A**) for 8 h or cells were left untreated. Cell lysates were submitted to the dual luciferase assay and relative luciferase units (RLU) were determined by normalizing firefly luciferase (ff) to the renilla luciferase (rnl) units of cotransfected cells. Data represent the mean ± SD from four independent experiments; (* *p* < 0.03 compared to no treatment). The structure of the two promoter constructs and the enhancer element from intron 1 including the known binding sites for the indicated transcription factors is shown in Figure S3 in the Supplemental Information.

In the next set of experiments, we aimed to verify the effect of ERK1/2 activation on insulin-induced PD-L1 expression by means of luciferase assays with PD-L1 promoter constructs. As shown in Figure 6A, in A818-6, T3M4, and BxPc3 cells, two different luciferase constructs were tested for their inducibility by insulin. When containing a 1.3 kb region of the 5′flanking region from the human PD-L1 gene (construct P1, see Figure S3 in the Supplemental Information), insulin treatment only slightly induced luciferase expression (Figure 6A). For comparison, IFNγ strongly activated this 1.3 kb promoter, as expected from the presence of three IRF binding sites within this gene region [43].

A same outcome was noticed when analyzing a 450 bp PD-L1 promoter fragment (construct P3, see Figure S3 in the Supplemental Information) that still contained one IRF binding site but lacked Nrf2 and AP-1 inducible elements, whereas a further shortened gene fragment (206 bp) also did not confer promoter activity to IFNγ (data not shown).

Thus, even though the longer promoter regions contain AP-1, STAT2/5, and STAT1/3 responsive elements (Figure S3) that are expected to be controlled by the insulin-induced signaling network [43,44,45,46], they were not sufficient for promoter activation under these conditions. As reported recently, there is an efficient enhancer element in the first intron of the PD-L1 gene [47,48], that strongly augments ERK1/2-inducible and AP-1-mediated PD-L1 gene promoter activation. In fact, when linking this enhancer containing dual AP1 binding sites [47] to the 1.3 kb promoter (construct P1-Enh, see Figure S3 in the Supplemental Information), the inducibility of the luciferase gene by insulin greatly increased in all three cell lines (Figure 6B). By contrast, linking the enhancer to the 450 bp PD-L1 promoter fragment (construct P3-Enh, see Figure S3 in the Supplemental Information) did not yield an inducing effect of insulin on luciferase. Thus, these data indicate that the AP-1 enhancer element exerts its activity in cooperation with upstream AP-1 sites in the PD-L1 promoter as shown previously [48].

### 2.7. Insulin-Induced PD-L1 Expression in PDAC Cells Confers Suppressive Activity against CD8+ T-Cells

**Figure 7:**
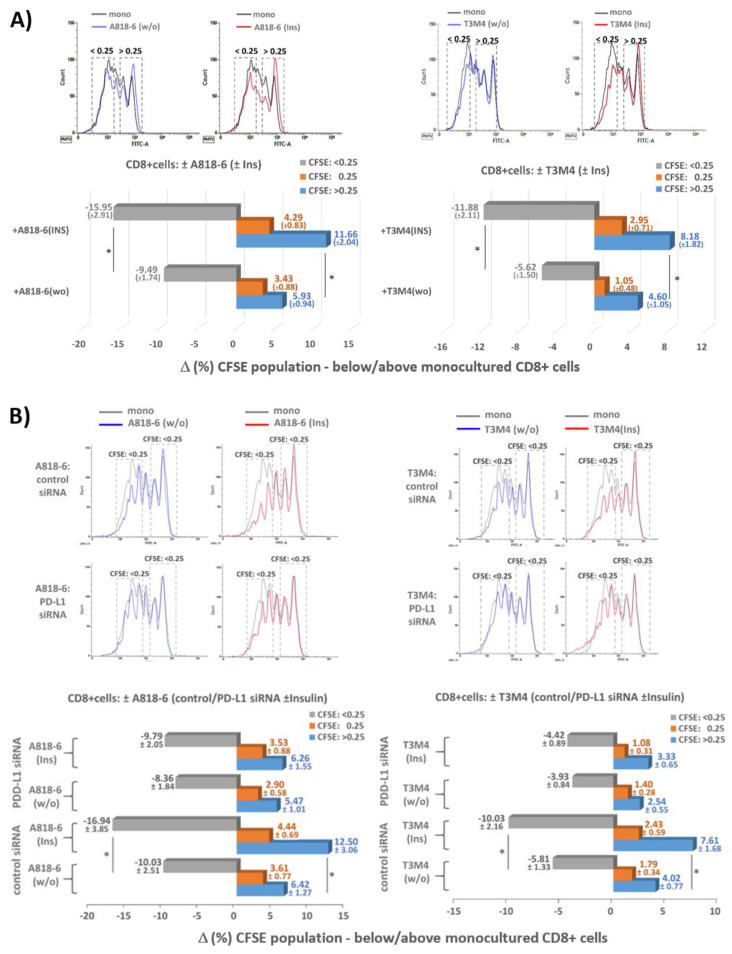

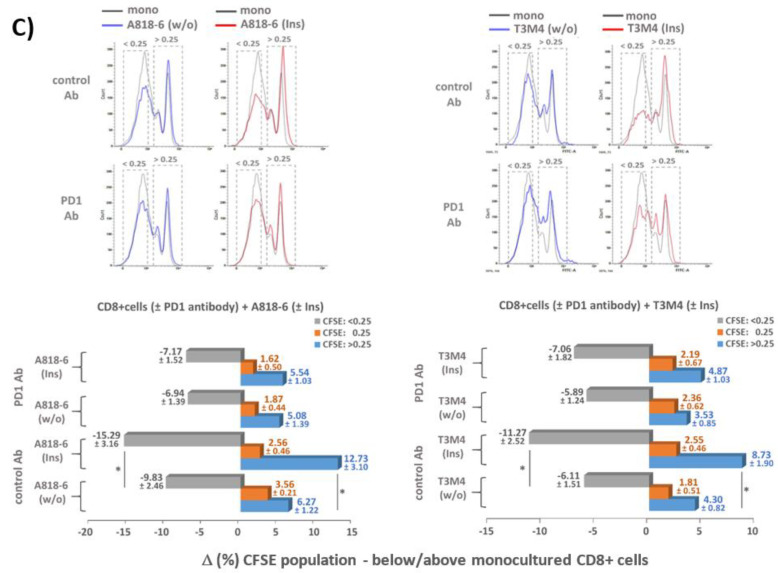
Insulin-induced PD-L1 expression in PDAC cells suppresses proliferative activity of CD8+ T-cells. (**A**) 1 × 10^5^ serum-starved A818-6 and T3M4 cells were left untreated (w/o) or treated with 0.1 IU/mL insulin (Ins) for 24 h. Then, cells were cocultured with 250,000 CFSE labelled and preactivated human CD8+ T-cells. At a final FCS concentration of 1% (*v*/*v*), PDAC and CD8+ T-cells were cocultured for 72 h. In parallel, CD8+ T-cells were monocultured under the same conditions. Afterwards, CD8+ T-cells were collected and submitted to flow cytometry for quantification of CFSE staining intensity. (**B**) A818-6 and T3M4 cells were pretreated with control or PD-L1 siRNA for 24 h, followed by serum starvation and subsequent treatment with 0.1 IU/mL insulin (Ins) or without (w/o) for 24 h. Then, CD8+ T-cells co/monoculture and flow cytometry for quantification of CFSE staining intensity were performed as described above. (**C**) Serum-starved A818-6 and T3M4 cells were left untreated or treated with 0.1 IU/mL insulin for 24 h. Then, the PDAC cells were cocultured with CFSE labelled and preactivated human CD8+ T-cells pretreated for 1 h with 2.5 µg/mL of PD-1 antibody (Pembrolizumab) or control antibody (Human IgG4, kappa). CD8+ T-cells co/monoculture and flow cytometry for quantification of CFSE staining intensity were performed as above. (**A**–**C**) The bar diagrams represent the percent decrease (−%) or increase (+%) of the three CFSE staining intensity fractions (<0.25, 0.25, >0.25) compared to monocultured CD8+ T-cells. The intensity fractions are highlighted by the dashed boxes in the representative histograms depicted in the upper panels. Data represent the mean ± SD from four (**A**,**B**) and five (**C**) independent experiments, respectively, performed in duplicates; (* *p* < 0.05 compared to untreated).

Next, we explored the functional effect of insulin-induced PD-L1 expression in coculture experiments with CD8+ T-cells. For this purpose, A818-6 and T3M4 cells that exhibit the strongest PD-L1 inducing effect by insulin (see above) were directly cocultured with preactivated (anti CD28/CD3 + IL-2 treatment for 4 days) and carboxyfluorescein succinimidyl ester (CFSE) labeled human CD8 + T-cells. When analyzing the CFSE staining intensity of the CD8+ T-cells by flow cytometry (Figure 7A) it was seen that compared to monocultured T-cells, those cocultured with A818-6 or T3M4 cells for 3 days showed a significant shift from less CFSE stained (intensity <0.25-fold = greater proliferation) towards higher CFSE-stained fractions (>0.25-fold = reduced proliferation). This shift by the coculture was even more pronounced when both PDAC cell lines were preincubated with insulin for 24 h, fitting together with the increased PD-L1 expression under this condition (see above and Figure S4 in The Supplemental Information).

To demonstrate PD-L1 dependency of the suppressive effect of A818-6 and T3M4 cells on the proliferation of cocultured T-cells, both cell lines were pretreated with PD-L1 siRNA. As shown in Figure 7B, the shift of CFSE staining in cocultured T-cells was less pronounced when exposed to the PDAC cells subject of PD-L1 knock-down (verified by Western blot, see Figure S5 in the Supplemental Information). In particular, the impact of the PD-L1 siRNA was most pronounced in insulin-treated T3M4 and A818-6 cells, as it can be appreciated from the greater fraction of cocultured T-cells displaying lower CFSE staining intensity (Figure 7B). We next investigated whether blocking of PD-1 is also able to reduce the inhibiting effect of insulin-pretreated PDAC cells on cocultured T-cells, since activated CD8+ T-cells exhibit considerable PD-1 expression. For this purpose, cocultures were carried out in the presence of the PD-1-blocking antibody Pembrolizumab (2.5 µg/mL). As shown in Figure 7C, similar to the observations made after PD-L1 knockdown in PDAC cells, the shift of CFSE intensity was much less pronounced in CD8+ T-cells after coculture with A818-6 and T3M4 cells in the presence of the PD-1 antibody as compared to the coculture in the presence of a control antibody. Again, the effect of the PD-1 antibody was most prominent in the cocultures with insulin-prestimulated A818-6 and T3M4 cells. These data indicate that insulin-induced PD-L1 expression in PDAC cells leads to inhibition of CD8+ T-cell proliferation.

### 2.8. Colocalization of Cytoplasmic (Activated) Insulin Receptor and PD-L1 in Human PDAC Tissue

**Figure 8:**
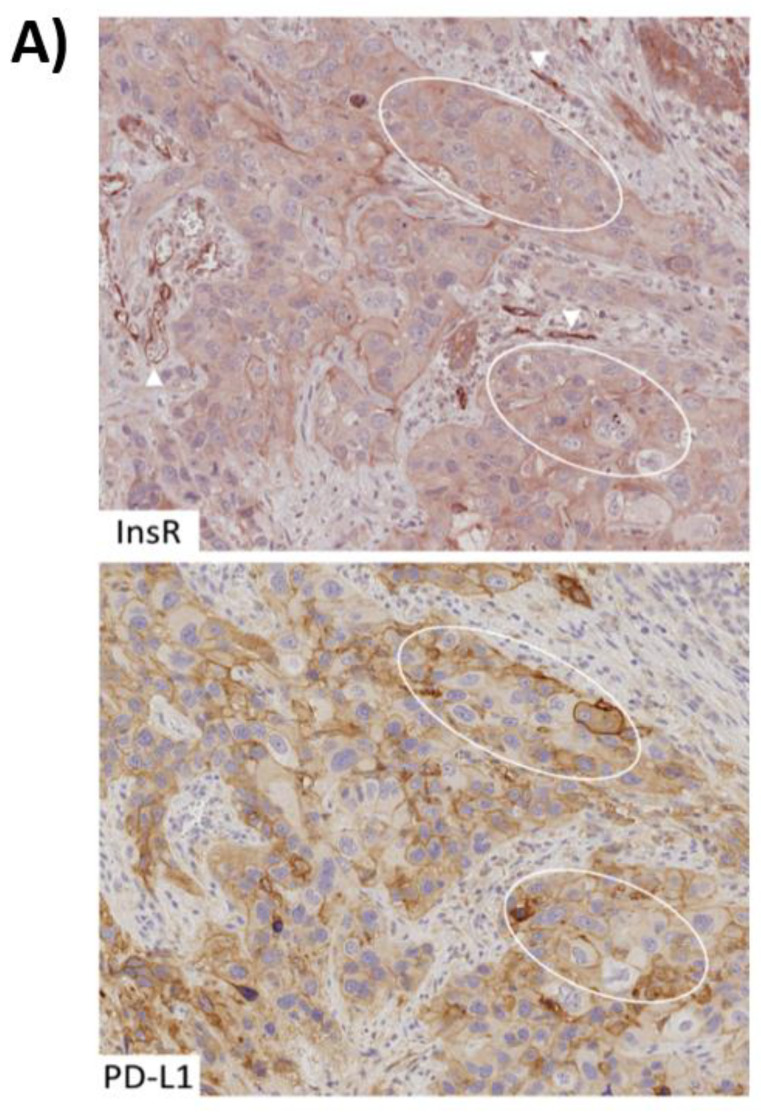

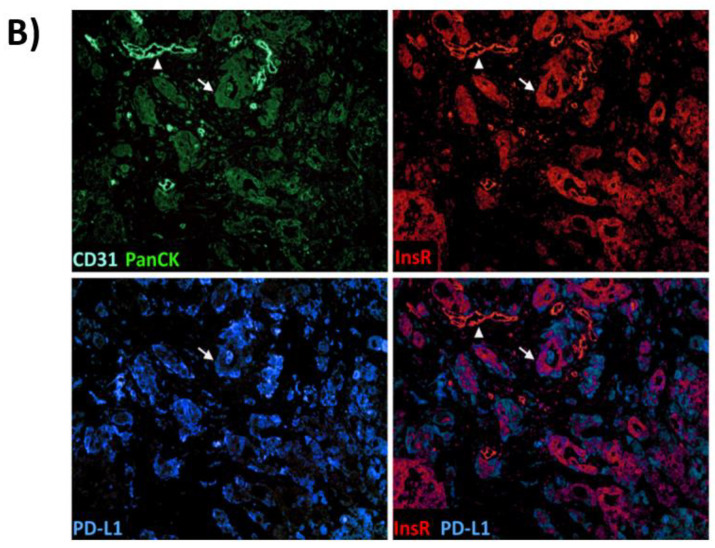
Colocalization of cytoplasmic (activated) insulin receptor and PD-L1 in human PDAC tissue. (**A**) Consecutive tissue sections of FFPE tissue from human PDAC samples were submitted to conventional immunostaining using an InsR and a PD-L1 antibody. Strongly membranous PD-L1-expressing areas regularly matched those with cytoplasmic InsR expression—two of which are depicted (marked by the ovals) and weaker PD-L1-expressing areas matched membranous InsR staining. Strongly (2+) InsR-expressing tumor vasculature is observed in the proximity (arrowheads). Original magnification: 200× (**B**). To validate PD-L1 and InsR colocalization in the context of tumor cells (arrow) and vasculature (arrowhead), immunofluorescence staining was performed. PanCK and CD31 are colored in green and turquoise, and InsR and PD-L1 are shown in red and blue, respectively (artificial color rendering). Images of InsR and PD-L1 expression are digitally merged to confirm colocalization (yielding purple). Original magnification: 100×.

Finally, we explored the expression of PD-L1 by immunohistochemistry in InsR-expressing PDAC samples (Figure 8). InsR was expressed in cancer cells as well as in cancer vasculature (Figure 8A). The staining intensities of InsR differed between the specimens, ranging from strong (2+) InsR expression in the cytoplasm (0–88%) and at the membrane (0–100%) of cancer cells, to weak (1+) cytoplasmic (0–100%) and membranous (0–70%) InsR expression. Areas devoid (0) of membranous and cytoplasmic InsR expression within the specimen varied between 0–95% and 0–70%, respectively. The extent of vascular InsR expression varied between the respective specimens, ranging from strong (2+) expression (0–100%), to weak (1+) expression (0–90%) or lacking (0) vascular InsR expression (0–100%).

PD-L1 expression was found in cancer cells and tumor infiltrating immune cells, mostly revealing faint and occasional immunostaining. In three (3.6%) of the analyzed InsR-expressing PDAC cases (*n* = 83), a pattern of intense PD-L1 expression in cancer cells was detected (Figure 8A). It indicates that this particular condition contributes to a PD-L1 immune checkpoint in a small subgroup of PDAC patients [32]. The tumor proportion scores (TPS) of the three cases were 50%, 80%, and 95%, respectively. Cytoplasmic InsR expression in PD-L1-positive tissue areas (Figure 8A) predominated membranous InsR expression (see Table 1). Intriguingly, vascular InsR expression was found to be particularly encompassing and intense within all three PD-L1-expressing PDAC samples.

To demonstrate colocalization of PD-L1 and InsR in the cancer cells, immunofluorescence was carried out using PD-L1 and InsR antibodies (Figure 8B). Concomitant immunofluorescence staining with PanCK and CD31 antibodies generally mapped all tumor cells as well as vessels within the region of interest. As it can be appreciated from Figure 8B, colocalization of PD-L1 and InsR was observed in the tumor cells but not in the tumor vasculature. Furthermore, InsR expression was visible in the cytoplasm of tumor cells indicating InsR activation by the insulin/IGF axis. Intriguingly, InsR staining regularly colocalized with PD-L1, thus underlining the association of the insulin/IGF axis with PD-L1-mediated immune control. We retrospectively assessed the T2DM status for the three InsR-expressing PD-L1-positive PDAC patients. Clinical data were available for only two out of three, with none of the respective patients having been documented to suffer from T2DM. HbA1c or fasting glucose values were not available for those patients.

## 3. Discussion

Modern immunological therapy concepts have opened new options for cancer treatment with immune checkpoints inhibitors. Tumors like PDAC, which rapidly progress and resist conventional therapies so far, were expected to be more efficiently treated with checkpoint inhibitors targeting the PD-1/PD-L1 pathway [30,31,36,37,49]. However, clinical trials in PDAC patients have been disappointing so far [38]. This could be explained by the fact that the role of the PD-1/PD-L1 system in immune evasion of PDAC cells has not been sufficiently elucidated yet, and thereby the suitability of PD-1/PD-L1-based immune therapies is not fully proven [37,38]. In our present study, we identified insulin as a potential trigger of the PD-1/PD-L1 axis based on its profound inducing effect on PD-L1 expression in PDAC cells. This finding sheds new light on the impact of hyperinsulinemia in the context of T2DM as a PDAC risk factor.

Using a panel of PDAC cell lines, we show that insulin directly induces PD-L1 expression through activation of the ERK pathway. Since insulin can interact with two types of the insulin receptor, InsR-A and InsR-B, both as homo or heterodimers, as well as with the IGF receptors, it creates a complex signalling network together with IGF1 and IGF2 [15] called the insulin/IGF axis. Thus, the inducing effect of insulin on PD-L1 expression is greatly determined by the insulin/IGF axis. In accordance with the complexity of this axis, the mechanisms by which insulin exerts its effect on PD-L1 varies between these PDAC lines. Thus, the most efficient induction of PD-L1 by insulin was seen in T3M4 cells depending on both InsR-A and IGFR, which are highly expressed in this cell line. The InsR-A/IGFR receptor configuration strongly activates the ERK pathway after binding to insulin, and the efficient induction of PD-L1 expression under this condition involves a potent enhancer element within the first intron of the PD-L1 gene [47]. In the PDAC cell line A818-6, which predominately expresses the InsR-B, insulin was less effective in inducing the ERK pathway and thereby PD-L1 expression. Likewise, BxPc3 cells that mainly express IGFR and only little InsR responded less to insulin in inducing ERK activation and PD-L1 expression. Since the insulin/IGF axis is connected to other growth factor signaling networks, we further explored the crosstalk of insulin and EGF. In line with previous findings [33], we observed an enhancing effect of EGF on insulin-induced PD-L1 expression.

Whilst the promoting effect of EGF on PD-L1 surface expression, as reported previously for colon cancer cells [33], seems to manifest in T3M4 cells, this mechanism is partially or even not involved in BxPc3 and A818-6 cells, respectively. In contrast to the reported posttranslational effect of EGF on PD-L1 expression that operates more additive to the induced expression of PD-L1 by insulin and depends on its forced trafficking to the cell surface [33], two other mechanisms in these two PDAC cell lines are involved (see Figure 4). Thus, we demonstrated that EGF promotes the inducing effect of insulin on PD-L1 in these PDAC cells already at the transcriptional level and in relation to an enhanced PD-L1 promoter activation. Interestingly, only the InsR-A-negative A818-6 and BxPc3 cells revealed this enhancing effect of EGF whereas the strongly InsR-A-expressing T3M4 cells did not, though being responsive to EGF, as seen by an altered phospho-ERK1/2 level. We could further show that EGF enhances InsR-A expression in A818-6 cells (see Figure 5), thereby altering the insulin/IGF axis to be more effective in terms of the insulin-induced P-ERK activation and thereby PD-L1 expression. Such an isotype switch from InsR-B to InsR-A has been previously reported in hepatocellular carcinoma cells [39]. In BxPc3 cells, EGF-induced InsR-A expression was less pronounced as these cells generally revealed very low InsR expression. Instead, BxPc3 cells showed a high expression of the Gab1 adaptor protein that essentially contributes to the crosstalk between EGFR signaling and the insulin/IGF axis [40]. Thus, Gab1 recruitment by the EGFR modulates the signaling of InsR and IGFR towards an enhanced ERK activation [40] and thereby stronger PD-L1 expression.

Depending on this complex crosstalk within the EGF/Insulin-IGF axis and the presence of the InsR-A, insulin is a strong inducer of PD-L1 expression in PDAC cells. This effect could be greatly influenced by the availability of the enhancer element in the PD-L1 gene (see above) that may be part of the methylation-dependent control of PD-L1 gene expression. In support of this, it was recently reported that differential methylation of promoters and enhancers define responders and non-responders to PD-1 inhibitors in non-small cell lung cancer patients [50]. Given such a scenario in PDAC, it can be speculated that demethylation of the PD-L1 enhancer and its accessibility to the KRAS/ERK pathway [48] may allow a forced expression of PD-L1 by insulin, thereby conferring a potent mechanism to PDAC cells by which they efficiently suppress the activity of CD8+ T-cells. Through this mechanism, higher insulin concentrations within the pancreatic organ could favor immune evasion by the tumor during PDAC development and progression.

Given the potential of insulin to induce PD-L1-mediated immune checkpoint control, as shown by our experiments, it was surprising to identify only few cases in the analyzed cohort of patients suffering from advanced PDAC that exhibited this connection between insulin and the PD-L1 immune checkpoint. One explanation could be the incomplete datasets in our retrospective study. Moreover, it can be speculated that the role of hyperinsulinemia manifests much earlier in pancreatic carcinogenesis, especially when keeping in mind that the PD-L1-dependent immune escape is more favorable during the onset of malignant cell growth. Under such condition, a T2DM along with elevated, intrapancreatic, interstitial insulin concentrations [51,52] would impact tumor development more severely. Moreover, a hidden and thereby nontreated T2DM that may develop shortly before pancreatic carcinogenesis could represent a particular risk constellation. With this in mind, forthcoming studies are needed to explore the link between insulin and PD-L1 in premalignant PanIN lesions and early PDAC. Recent data indicated that hyperinsulinemia promotes PanIN formation in Kras^G12D^ mice, an effect relying on the mitogenic activity of insulin [53]. However, the association of hyperinsulinemia with the PD-L1 immune checkpoint still remained to be investigated. Thus, the usage of Kras^G12D^ mice subjected to a diabetogenic diet high in fats and calories, which develop PDAC [54], would be an appropriate model in vivo to establish the link between hyperinsulinemia and PD-L1 checkpoint control.

In addition, forthcoming studies should enroll larger cohorts of PDAC patients with a clear T2DM history on the one hand and prediabetic hyperinsulinemic PDAC patients on the other hand, as well as non-diabetic PDAC patients serving as the control group. Nevertheless, a diabetic risk constellation and T2DM history could serve as a criterion for stratifying PDAC patients to apply PD-1/PD-L1 checkpoint inhibitors such as Pembrolizumab or Atezolizumab for immune therapy that otherwise may fail due to the absence of PD-L1 expression in many PDAC cases (see above) [32,38,55,56]. Indeed, those PDAC tissue areas strongly expressing PD-L1 displayed cytoplasmic InsR staining, as shown above (Figure 8). Thus, if present in PDAC, PD-L1-dependent immune evasion seems to be linked to the activation of the insulin/IGF axis. The impact of insulin on PD-L1 mediated immune evasion of PDAC also represents a novel pathophysiological mechanism that provides another rational for using anti-diabetic medications like metformin [57,58] also in the treatment of PDAC, thereby abrogating the immune privilege of PDAC cells due to hyperinsulinemia.

Finally, and maybe most importantly, a main challenge for PD-L1 inhibition in PDAC lies in the selection of suitable patients. PD-L1 has been described to be a dynamic biomarker [59] and its expression can fluctuate over the course of time. This is particularly driven by changes in the local tumor microenvironment, including an alternating exposure to insulin. The absence of PD-L1 expression in an acquired sample can therefore be a false negative finding due to the time point the biopsy was taken [59,60]. Based on the inducibility of PD-L1 by insulin, as reported here, it can be speculated that when the biopsy is taken or a surgery is performed, patients are usually in a fasting state and hence their insulin levels are comparatively low. As a practical clinical approach, in order to account for insulin-mediated PD-L1 inducibility, a moderately dosed glucose infusion before the biopsy or surgery of the fasting patient would trigger insulin secretion and might reveal an otherwise undetectable PD-L1 expression. This approach should be evaluated in future studies, as it could potentially increase the predictive value of PD-L1 diagnostics in biopsy specimens. Unveiling otherwise missed insulin-mediated PD-L1 expression by an adaptation of intervention protocols could lead to a more customized and therefore effective application of PD-L1 inhibitors.

## 4. Materials and Methods

### 4.1. Cell Lines and Culture

The human PDAC cell lines Panc1, MiaPaca2, and BxPc3 were obtained from the DSZM (Braunschweig, Germany). T3M4 cells were kindly provided by H. Friess (Heidelberg, Germany) and A818-6 cells were a gift from H. Kalthoff (Kiel, Germany). Culture conditions were as described recently [61,62,63]. Cell line authenticity was checked by STR-profiling.

### 4.2. Stimulants and Inhibitors

Human Epidermal growth factor (EGF) was purchased from Upstate Biotechnology (Waltham, MA, USA), FR180204 from Sigma (St.-Louis, MO, USA), Bez235 from Cayman Chemicals (Ann Arbor, MI, USA), and IFNγ, IGF1, and IGF2 from ReliaTech (Wolfenbüttel, Germany). Regular (short acting) insulin was obtained from Berlin Chemie (Berlin, Germany).

### 4.3. RNA Preparation and Real-Time PCR

RNA isolation, reverse-transcription into cDNA, and real-time PCR (Biorad Cycler) using the SYBR-Green assay (Fermentas) were carried out as described [63]. All primers (MWG-Eurofins, Ebersberg, Germany) were used at a final concentration of 0.2 µM. Cycling conditions were: 95 °C for 7 min initial denaturation followed by 45 cycles at 95 °C for 5 s and 60 °C 30 s. The following primer sets were used: 5′-RPL13 forw/rev; 5′-gctgcactaatgttattggga3′/5′aattcgcttgtagtcggcacc-3′; PD-L1 forw/rev, 5′-cctggaggagaagagg-3′/5′ttgaggacctctgtgtatttgtcaa-3′, InsR-A forw, 5′-ttttcgtccccaggccatc-3′; InsR-B forw, 5′-ccccagaaaaacctcttcagg-3′; InsR rev, 5′-gtcacattcccaacatcgcc-3′.

### 4.4. Western Blotting

Total cell-lysates were prepared, separated by SDS-PAGE, and submitted to Western blotting as described [62,63]. Antibodies were used as follows: Anti-PD-L1 (cat. ab205921, Abcam, 1:1000 in Skim/TBST), anti-Hsp90 (cat. sc7947, 1:1000 in Skim/TBST), and anti-Gab1 (cat. sc133191, Santa Cruz, Heidelberg, Germany, 1:200 in Skim/TBST), anti-P-ERK1/2 (cat. 9101, 1:1000 in BSA/TBST), anti-P-AKT (cat. 9271, 1:2000 in BSA/TBST), anti-ERK1/2 (cat. 9102, 1:1000 in BSA/TBST), anti-AKT (cat. 9272, 1:2000 in BSA/TBST), anti-InsR (cat. 3025, 1:500 in BSA/TBST), anti-IGFR (cat. 14534, 1:500 in BSA/TBST), anti-rabbit HRP (cat 7074, 1:4000 in skim/TBST), and anti-mouse HRP (cat 7074, Cell Signaling Technology, Frankfurt, Germany, 1:4000 in skim/TBST), anti-αTubulin (cat. T5168, Sigma 1:10000, in Skim/TBST). Blots were analyzed with the ChemiDoc gel documentation system (Biorad, Munich, Germany). Relative band intensities were calculated by the QuantityOne software (Biorad) and the band densities were normalized to the corresponding housekeepers Hsp90 and tubulin or unphosphorylated Akt and ERK1/2, respectively.

### 4.5. siRNA Transfection

For siRNA (Qiagen, Hilden, Germany) transfection, cells grown in 12-well plates were lipofected (6 µL of HiPerfect-reagent, Qiagen) with 150 ng/well of control siRNA, InsR siRNA (no. SI00004515), IGF1R siRNA (no.SI02624552), and Gab1 siRNA (no.SI00031913). PD-L1 siRNA (cat. SC-39699) and the corresponding control (sc-37007) were purchased from Santa-Cruz (Heidelberg, Germany).

### 4.6. Generation of PD-L1 Promoter Constructs

Based on the recent characterization of the PD-L1 promoter [47], fragments of the PD-L1 promoter (depicted in Figure S3 in the Supplemental Information ) encompassing the positions -151444 to -152723 (GenBank: AL135786.17, Chr. 9p23-24.3) yielding the 1.28 kb (P1) construct and -152275 to -152723 (GenBank: AL135786.17, Chr. 9p23-24.3) yielding the 0.45 kb (P3) construct were generated by PCR from human genomic DNA using the forward primers 5′-gagaactccatgctcctgccaa-3′ and 5′-ggtaaaatcaaggtgcgttcag-3′, respectively, together with the reverse primer 5′-*gctagc*cagagatactgggcc-3′ (all from MWG-Eurofins, Ebersberg, Germany). In addition, a primer set incorporating a *Sal-I* restriction site was used (5′- cactctgacttccgtattcctc-3′/5′-*gtcgac*taacagtggggagtgggaa -3′) to amplify the potential 200 bp enhancer element -874 to -1094 (GenBank: AL152253.17, Chr. 9) within intron 1 of the PD-L1 gene [46,47]. After cloning into the pCR2.1-TA-Topo cloning vector (Thermo-Fisher Scientific), the promoter fragments P1 and P3 were ligated into the pGL3basic luciferase vector (Promega) via Kpn-I and Nhe-I restriction sites. The enhancer element was ligated into the empty pGL3-basic or the P1- and P3-pGL3 vectors through *Sal-I* and *BamH-I* sites. All constructs were checked by DNA sequencing.

### 4.7. Dual Luciferase Assays

Firefly luciferase expression controlled by PD-L1-promoter constructs was determined by triplicate measurements using the *Dual-Glow* luciferase assay (Promega, Mannheim, Germany). For normalization, constitutive *Renilla*-luciferase expression was determined thereafter.

### 4.8. Immunostaining of Unpermeabilized Cells and Flow Cytometry

Cells were collected, washed, and resuspended in MACS buffer (PBS with 5mM EDTA, 2% (*w*/*v*) BSA). Then, cells were stained with anti-PD-L1 antibody recognizing the N-terminus (Abcam ab205921, Cambridge, UK) at 1:200 in MACS buffer or the corresponding rabbit IgG control antibody for 1 h at room temperature. After extensive washing, cells were incubated with donkey-anti rabbit Alexa Fluor^®^ 488 (no A21206 Thermo Scienfitic, MA, USA) at 1:500 dilution for 1 h at room temperature, followed by one wash step and fixation in 1% (*v*/*v*) formaldehyde in MACS buffer. Immuno staining was analyzed by fluorescence flow cytometry on a FACSVerseTM instrument (Becton Dickinson, NJ, USA) using FACSuite Flow Cytometry Software (Becton Dickinson).

### 4.9. CD8-T-Cell Isolation, CFSE Staining and Activation

CD8+T-cells were isolated from the peripheral blood of healthy donors who gave written informed consent prior to the study. Cells enriched from the blood samples by counterflow elutriation were subjected to gradient centrifugation using Pancoll separation solution (Pan Biotech, Aidenbach, Germany). The separated interphase cells were collected and then subjected to red blood cell lysis using 155 mM NH4Cl, 10 mM KHCO3, 0.1 mM EDTA buffer. Then, cells were centrifuged (300× *g*, 10 min, 4 °C), resuspended in T-cell medium (Pan Biotech), and left in culture flasks for 1 h at 37 °C to remove adherent cells. Afterwards, nonadherent cells were collected and enriched for CD8+Tcells by MACS separation (CD8+ T-cell Isolation Kit from Miltenyi, Bergisch Gladbach, Germany) following the instructions by the manufacturer. CD8+ T-cells were checked for purity by anti CD4/CD8 and CD3 double staining and subsequent flow cytometry. Then, cells were stained with 10 µM CFSE (BioLegend, San Diego, USA) in T-cell medium for 10 min at 37 °C in the dark. Thereafter, CD8+ T-cells were washed twice in T-cell medium, counted and seeded in 12-well plates precoated with anti CD3 antibody (BioLegend), and at a density of 1.5 million/mL in T-cell medium (RPMI 1640 + 10% FCS) supplemented with 1.5 µg/mL anti-CD28 antibody (BioLegend) and 60 ng/mL IL-2 (BioLegend). After 3 d, IL-2 was added again.

### 4.10. Coculture of CD8+ T-Cells and PDAC Cells

A818-6 and T3M4 cells (1 × 10^5^) were seeded on 12-well plates in regular medium. After 1d, medium was replaced by RPMI 1640 + 1% FCS medium and 24 h later insulin and EGF were administered or not. After another 1d, medium was replaced by serum-free RPMI 1640, and 1/10 volume of activated CD8+ T-cell suspension in full medium was added to obtain a final T-cell density of 0.25 million/mL and a final FCS concentration of 1%. Cells were cocultured for 3d, then CD8+ T-cells were removed and collected in MACS buffer for flow cytometry. CFSE staining intensity was measured and the fractions along with the linear fluorescence intensities were quantified. For comparison, CFSE-stained and activated CD8+ T-cells that underwent monoculture for the same period were measured in parallel. To assess the shift in the single intensity fractions absolute differences in the percentage were calculated. To block PD-1 mediated T-cell suppression, in some experiments, an anti PD-1 antibody (Pembrolizumab (Keytruda^®^)) from MSD, Haar, Germany) or a control antibody (Human IgG4, kappa, Biovision, Biozol, Eching, Germany) was added at a concentration of 2.5 µg/mL for 1 h to the CD8+ T-cells before the coculture.

### 4.11. Patients and Tissues

From the archive of the Institute of Pathology, University Hospital Schleswig-Holstein, Kiel, we retrieved all tissues from patients with PDAC who had undergone a surgery (Whipple procedure) for PDAC resection or had received a diagnostic biopsy between 1999 and 2017. Only patients with histologically confirmed PDAC with InsR expression as confirmed by immunohistochemistry were included in the study. The study population was comprised of 83 patients (average age 67 years (40–85 years), 42 male and 41 female. All tumor stages were represented, with the majority of 61.4% having been classified as UICC stage IIB at the time of diagnosis).

### 4.12. Histology

Following fixation in neutral buffered formalin, all tissue specimens were embedded in paraffin. The specimens were sectioned, deparaffinized, and subsequently stained with hematoxylin and eosin. The World Health Organization criteria were used for histological classification. According to the 8th edition of the UICC guidelines, the pTNM-stage of all study patients was determined.

### 4.13. Immunohistochemistry and Evaluation of Immunostaining

InsR immunostaining was carried out manually. For InsR immunostaining a rabbit monoclonal anti-insulin receptor β-antibody (dilution 1:50; clone 4B8; Cell Signaling Technologies, Danvers, MA, USA) was used, which detects both InsR isoforms (further details see Supplemental Information). PD-L1 immunostaining was performed with monoclonal antibodies directed against PD-L1 (dilution 1:100; rabbit monoclonal antibody; E1L3N; Cell Signaling Technology) using the autostainer Bond™ Max System (Leica Microsystems GmbH, Wetzlar, Germany) according to the manufacturer’s instructions and as extensively described in the Supplemental Information. PD-L1 expression in tumor cells was evaluated using the tumor proportion score (TPS), which is defined as the percentage of cancer cells showing membranous PD-L1 staining. Cytoplasmic and membranous InsR expression in tumor cells as well as InsR expression in tumor vasculature were separately evaluated with regard to the intensity of immunostaining (0 = no evidence of staining; 1+ = weak staining; 2+ = strong staining) and the proportion of the respective staining intensity within a given sample [64].

### 4.14. Immunofluorescence

Immunofluorescence staining served to illustrate colocalization of InsR and PD-L1 expression in the PD-L- and InsR-expressing PDAC specimen. Immunofluorescence staining reagents were used from the Opal 7-color automated IHC kit (Akoya Biosciences, Marlborough, MA, USA), as well as from the Opal Polaris 780 reagent pack (Akoya Biosciences, Marlborough, MA, USA). The immunofluorescence staining procedure was performed with the autostainer Bond™ Max System (Leica Microsystems GmbH, Wetzlar, Germany). The tissue slide was stained with antibodies against PanCK (dilution 1:200; mouse monoclonal antibody; AE1/AE3; NeoMarkers via Thermo Fisher Scientific, Waltham, MA, USA), CD31 (dilution 1:100; mouse monoclonal antibody; 131M-16; Cell Marque, CA, USA), InsR β (dilution 1:50; rabbit monoclonal; clone 4B8; Cell Signaling Technologies, Danvers, MA, USA), and PD-L1 (dilution 1:100; rabbit monoclonal antibody; E1L3N; Cell Signaling Technologies, Danvers, MA, USA). Tissue slides were also stained with anti-MPO, anti-CD3, and anti-CD68 antibodies using the Opal 7-color automated IHC kit to map the local immune cell infiltration (data not shown). For further details see the Supplemental Information. The fluorescence microscope Nikon Eclipse Ni (Nikon Europe B.V., Amsterdam, Netherlands) was used for fluorescence microscopy and the full-spectrum ZWO Kamera ASI 183 MC Color (Suzhou ZWO CO., LTD., Sozhou, JiangSu Province, China) was used to capture the images.

### 4.15. Statistical Analysis

As indicated in the figure legends, normally distributed data were evaluated by the two-tailed Student’s *t*-test (using Excel 2013 run on Microsoft-Windows 8.1, Redmond, WA, USA) assuming equal variance (*p* < 0.05 is considered statistically significant). All data were included in statistical analysis with no randomization or blinding. No data points were excluded.

## 5. Conclusions

Type 2 diabetes, a disease characterized by insulin resistance and hyperinsulinemia, represents a considerable risk factor for PDAC. The inducing effect of insulin on PD-L1 expression in PDAC cells that is described in this study represents another important mechanism by which a diabetic and hyperinsulinemic condition could favor PDAC development and progression. Besides the well- known growth-promoting activity of insulin and the other members of the insulin/IGF axis, they also appear to play an essential role in tumoral immune escape mechanisms. Based on a better understanding of this insulin effect, strategies to interfere with it—for example with metformin—could pave the way towards a more efficient immunotherapy.

## Figures and Tables

**Table 1:** Characterization of PDAC specimens with tumoral PD-L1 and InsR expression. PD-L1 expression in tumor cells was evaluated using the tumor proportion score (TPS), which is defined as the percentage of cancer cells showing membranous PD-L1 staining. Cytoplasmic and membranous InsR expression in tumor cells were separately evaluated with regard to the intensity of immunostaining (0 = no evidence of staining; 1+ = weak staining; 2+ = strong staining). The proportion of InsR-expressing tumor cells within a given sample is given in percent (%).

Case No.	TPS	Cytoplasmic InsR Expression	Immunostaining Intensity 1+	Immunostaining Intensity 2+	Membranous InsR Expression	Immunostaining Intensity 1+	Immunostaining Intensity 2+
1	50%	98%	68%	30%	55%	35%	20%
2	80%	95%	90%	5%	85%	60%	25%
3	95%	100%	95%	5%	16%	15%	1%

## Data Availability

All data generated and analysed during this study are included in this published article.

## Supplementary Materials

The following are available online at https://www.mdpi.com/article/10.3390/medsci9030048/s1, Additional results on time and dose dependency of insulin inducible PD-L1 expression and additional methodology/Figure S1: Time and dose dependency of the effect of Insulin on PD-L1 expression in PDAC cells/Figure S2: Validation of AKT inhibition by Bez235 and ERK1/2 inhibition by FR180204 in PDAC cells/Figure S3: Structure of PD-L1 gene promoter constructs and the enhancer element from intron 1/Figure S4: Insulin-induced PD-L1 expression in A818-6 and T3M4 cell is maintained during T-cell cocultures/Figure S5: Knock down of PD-L1 expression in PDAC cells during T-cell coculture experiments.

## Abbreviations

CFSE, carboxyfluorescein succinimidyl ester; EGF, epidermal growth factor; ERK-1/2, extra-cellular response kinase 1/2; Gab1, Grb2 associated binder 1; IFNγ, Interferone gamma; IGF, insulin like growth factor; Ins, Insulin; IU, International Unit; PanIN: Pancreatic Intraepithelial Neoplasia; PDAC, pancreatic ductal adenocarcinoma; PD-1, Programmed death protein-1; PD-L1, Programmed death-ligand 1; T2MD, type2 diabetes mellitus.
