# Supplementary material for: Programmed Death-Ligand 1 (PD-L1) Expression Is Induced by Insulin in Pancreatic Ductal Adenocarcinoma Cells Pointing to Its Role in Immune Checkpoint Control
Source: Med Sci (Basel). 2021 Jun 25;9(3):48. doi: 10.3390/medsci9030048 (PMC8293454; doi:10.3390/medsci9030048)
Supplement: Supplementary file 1 [file medsci-09-00048-s001.zip › medsci-1176503-supplementary.pdf]

Article

Programmed Death-Ligand 1 (PD-L1) Expression Is Induced by Insulin in Pancreatic Ductal Adenocarcinoma Cells Pointing to its Role in Immune Checkpoint Control

Steffen M. Heckl [1,2], Franziska Mau [3], Anke Senftleben [3], Tina Daunke [3], Silje Beckinger [3], Samir Abdullazade [4], Stefan Schreiber [1], Christoph Röcken [4], Susanne Sebens [3] and Heiner Schäfer [1,3,*]

[1] Department of Internal Medicine I, UKSH Campus Kiel, Arnold-Heller-Str. 3, Bldg. K3, 24105 Kiel, Germany; steffen.heckl@uksh.de (S.M.H.); stefan.schreiber@uksh.de (S.S.)
[2] Department of Internal Medicine II, UKSH Campus Kiel, university, Arnold-Heller-Str. 3, Bldg. E,
24105 Kiel, Germany
[3] Institute of Experimental Cancer Research, UKSH Campus Kiel & Christian-Albrechts-University Kiel, Arnold-Heller-Str. 3, Bldg. U30, 24105 Kiel, Germany; franziska-mau@gmx.de (F.M.); as.senftleben@googlemail.com (A.S.); tina.daunke@email.uni-kiel.de (T.D.); silje.beckinger@email.uni-kiel.de (S.B.); susanne.sebens@email.uni-kiel.de (S.S.)
[4] Department of Pathology, Christian-Albrechts-University Kiel, Arnold-Heller-Str. 3, Bldg. U33,
24105 Kiel, Germany; samir.abdullazade@uksh.de (S.A.); christoph.roecken@uksh.de (C.R.)
* Correspondence: hschaef@1med.uni-kiel.de; Tel.: +49-(431)-500-22411

Supplemental Information

2. Results

2.1. Insulin inducible PD-L1 expression in PDAC cells.

Next, we could demonstrate that the effect of insulin in the three responsive cell lines was time-dependent. As shown by Western blot analysis (Figure S1A), the induction of PD-L1 protein expression by insulin was most pronounced in A818-6 and BxPc3 cells after 24 h and 48 h, respectively, whereas in T3M4 cells the insulin mediated induction of PD-L1 expression became visible already after 8 h (Figure S1A). Likewise, the increase of PD-L1 mRNA upon administration of 0.1 IU/mL insulin was higher in T3M4 cells after 8 h (8,74-fold) than after 32 h (5.92-fold) whereas in A818-6 cells the insulin induced PD-L1 mRNA expression was more delayed revealing an increase of 5.3-fold and 9.84-fold after 8 h and 32 h, respectively (Figure S1B). In BxPc3 cells, insulin treatment induced PD-L1 mRNA expression only moderately after both periods (2.71-fold and 3.64-fold after 8 h and 32 h, respectively).
Furthermore, the insulin mediated PD-L1 protein expression was also dose dependent (Figure S1 C+D). At a dose of 0.1 IU/mL, insulin exerted the greatest effect on PD-L1 expression in A818-6 and BxPc3 cells, whereas in T3M4 cells already a lower concentration (0.02 IU/mL) of insulin induced PDL1 expression alomst maximally. At a higher dose of 0.5 IU/mL, the effect on PD-L1 expression by insulin was less pronounced in these three cell lines. This could be due to a forced InsR downregulation and desensitization at this higher insulin dose.

4. Materials & Methods

4.4. Western blotting.

Total cell-lysates were prepared, separated by SDS-PAGE and submitted to western blotting. Antibodies were use as follows: anti-P(Ser727)-STAT3 (cat. 9101, 1:1000 in BSA/TBST), anti-P(Ser21/9)-GSK3α/β (cat. 9271, 1:2000 in BSA/TBST) and anti-STAT3 (cat. 9102, 1:1000 in BSA/TBST), GSK3α/β (cat. 9272, 1:2000 in BSA/TBST) from Cell Signalling (Frankfurt, Germany). Blots were analyzed with the ChemiDoc gel documentation system (Biorad, Munich, Germany). Relative band intensities were calculated by the QuantityOne software (Biorad) and the band densities were normalized to the corresponding house keepers Hsp90 or tubulin

4.12. Immunohistochemistry.

InsR immunostaining was carried out manually. For InsR immunostaining a rabbit monoclonal anti-insulin receptor β-antibody (dilution 1:50; clone 4B8; Cell Signaling Technologies, Danvers, USA) was used, which detects both IR isoforms. Following deparaffinization, all sections were boiled in EDTA buffer (pH 9.0; 1 min; 125 °C), then washed with Tris-buffered saline (TBS) and then treated with hydrogen peroxide block (Thermo Fisher Scientific) for 15 min, washed with TBS and then blocked with Ultra V Block (Thermo Fisher Scientific) for 5 min. The ImmPRESS reagent peroxidase universal anti-mouse/rabbit Ig – MP-7500 and the ImmPact NovaRed peroxidase substrate SK-4805 Kit (Vector Laboratories, Burlingame, CA, USA, respectively) were used for the visualization of immunoreactions. Subsequently, a counterstaining with hematoxylin was carried out. The omission of the primary antibody served for negative controls. Healthy endometrium samples (proliferative phase) were used as positive controls.

PD-L1 immunostaining was performed with monoclonal antibodies directed against PD-L1 (dilution 1:100; rabbit monoclonal antibody; E1L3N; Cell Signaling Technologies, Danvers, USA) using the autostainer Bond™ Max System (Leica Microsystems GmbH, Wetzlar, Germany) according to the manufacturer`s instructions. Antigen retrieval was carried out with the ER2 buffer (EDTA-buffer Bond pH 9.0). The Bond™ Polymer Refine Detection Kit (DS 9800; brown labelling; Novocastra; Leica Microsystems GmbH, Wetzlar, Germany) was employed for the immunoreaction. A PD-L1 expressing cancer sample was employed as a positive control.

4.14. Immunofluorescence.

Immunofluorescence staining served to illustrate colocalization of InsR and PD-L1 expression in an exemplary PD-L1 and InsR expressing PDAC specimen. Immunofluorescence staining reagents were used from the Opal 7-color automated IHC kit (Akoya Biosciences, Marlborough, USA), as well as from the Opal Polaris 780 reagent pack (Akoya Biosciences, Marlborough, USA). In brief, the Opal system is based on the tyramide signal amplification (TSA) staining principle, which utilizes HRP (horseraddish peroxidase)-linked secondary antibodies and HRP-activated heat-stable TSA-fluorophores. Effective heat-induced stripping of primary and secondary antibodies allows conducting successive immunofluorescence staining cycles, even when using primary antibodies from the same species.

The immunofluorescence staining procedure was performed with the autostainer Bond™ Max System (Leica Microsystems GmbH, Wetzlar, Germany). The autostainer Bond™ Max System's software had originally not been designed for the Opal staining procedure, which is intended to be used with the Bond™ RX autostainer system. Therefore the Opal automation staining protocol published by Akoya Biosciences, Marlborough, USA, had to be manually implemented and slightly modified in order to be accepted by the underlying software: Successful implementation of the procotol requires adding a second peroxidase blocking reaction step before each staining procedure using the peroxidase blocking reagent of the Bond™ Polymer Refine Detection Kit (DS 9800; brown labelling; Novocastra; Leica Microsystems GmbH, Wetzlar, Germany).

The tissue slide was stained with antibodies against PanCK (dilution 1:200; mouse monoclonal antibody; AE1/AE3; NeoMarkers via Thermo Fisher Scientific, Waltham, USA), CD31 (dilution 1:100; mouse monoclonal antibody; 131M-16; Cell Marque, California, USA), InsR β (dilution 1:50; rabbit monoclonal; clone 4B8; Cell Signaling Technologies, Danvers, USA) and PD-L1 (dilution 1:100; rabbit monoclonal antibody; E1L3N; Cell Signaling Technologies, Danvers, USA). Antigen retrieval was done with ER2 (EDTA buffer Bond pH 9.0). The tissue slide was also stained with anti-MPO, anti-CD3 and anti-CD68 antibodies using the Opal 7-color automated IHC kit to map the local immune cell infiltration.

The PanCK- and CD31- staining was performed simultaneously by preparing a mixed primary antibody solution containing both antibodies with the dilution factors referenced above and with Opal 520 (dilution 1:150) serving as the common fluorophore. InsR was visualized using the Opal 620 fluorophore (dilution 1:150). PD-L1 was stained with the Opal 780 fluorophore, which necessitates an additional amplification step according to the manufacturer's instructions: The binding of the HRP-labeled secondary antibody is followed by a HRP-catalyzed covalent deposition of digoxigenin (DIG; Opal TSA-DIG working solution 1:50), which in turn is bound by the fluorescently labelled anti-DIG-antibody Opal Polaris 780 (Opal Polaris 780 working solution 1:25). Since this dye emitts in the near infrared range, the signal from PD-L1 immunostaining was pseudo-colored in blue. The slide was mounted with ProLong™ Diamond Antifade mounting medium (Thermo Fisher Scientific, Waltham, USA). The fluorescence microscope Nikon Eclipse Ni (Nikon Europe B.V., Amsterdam, The Netherlands) was used for fluorescence microscopy and the full-spectrum ZWO Kamera ASI 183 MC Color (Suzhou ZWO CO., LTD., JiangSu Province, China) was used to capture the images.

Figures S1 - S15

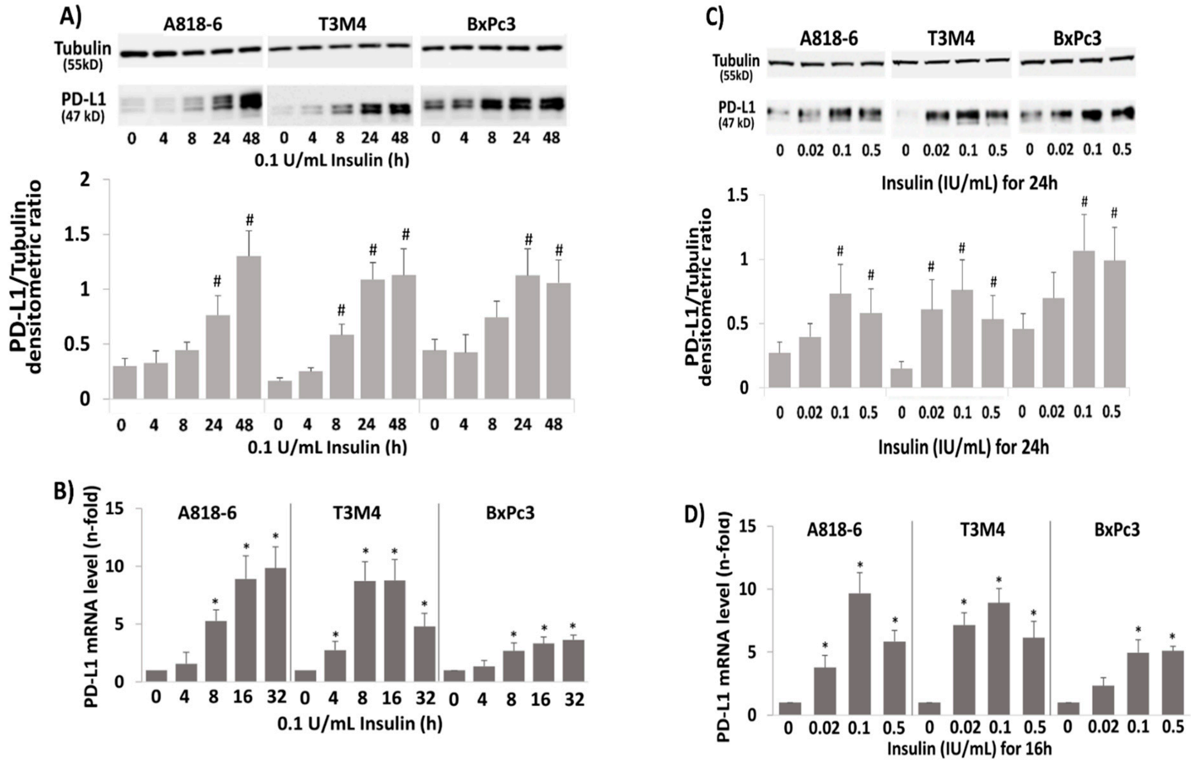

Figure S1. Time and dose dependency of the effect of Insulin on PD-L1 expression in PDAC cells. Serum starved A818-6, T3M4 and BxPc3 cells were **A)** and **B),** either left untreated or were treated with 0.1 IU/mL insulin for the indicated periods, or were **C)** and **D)**, either left untreated or were treated with increasing amounts (0.02, 0.1 and 0.5 IU/mL) of insulin for C) 24 h and D) 16h. **A)** and **C)** Western blot analyses using PD-L1 and Tubulin antibodies were performed. Representative blots from 4 independent experiments and band densitometry analyses (lower panels, #p<0.05, $n = 4$) are shown. **C)**, and **D),** qPCR analysis using PD-L1 and RPL13 primers. PD-L1 expression is normalized to the expression level of RPL13 and data represent the mean ± SD from 4 independent experiments (*p<0.03 compared to un-treated).

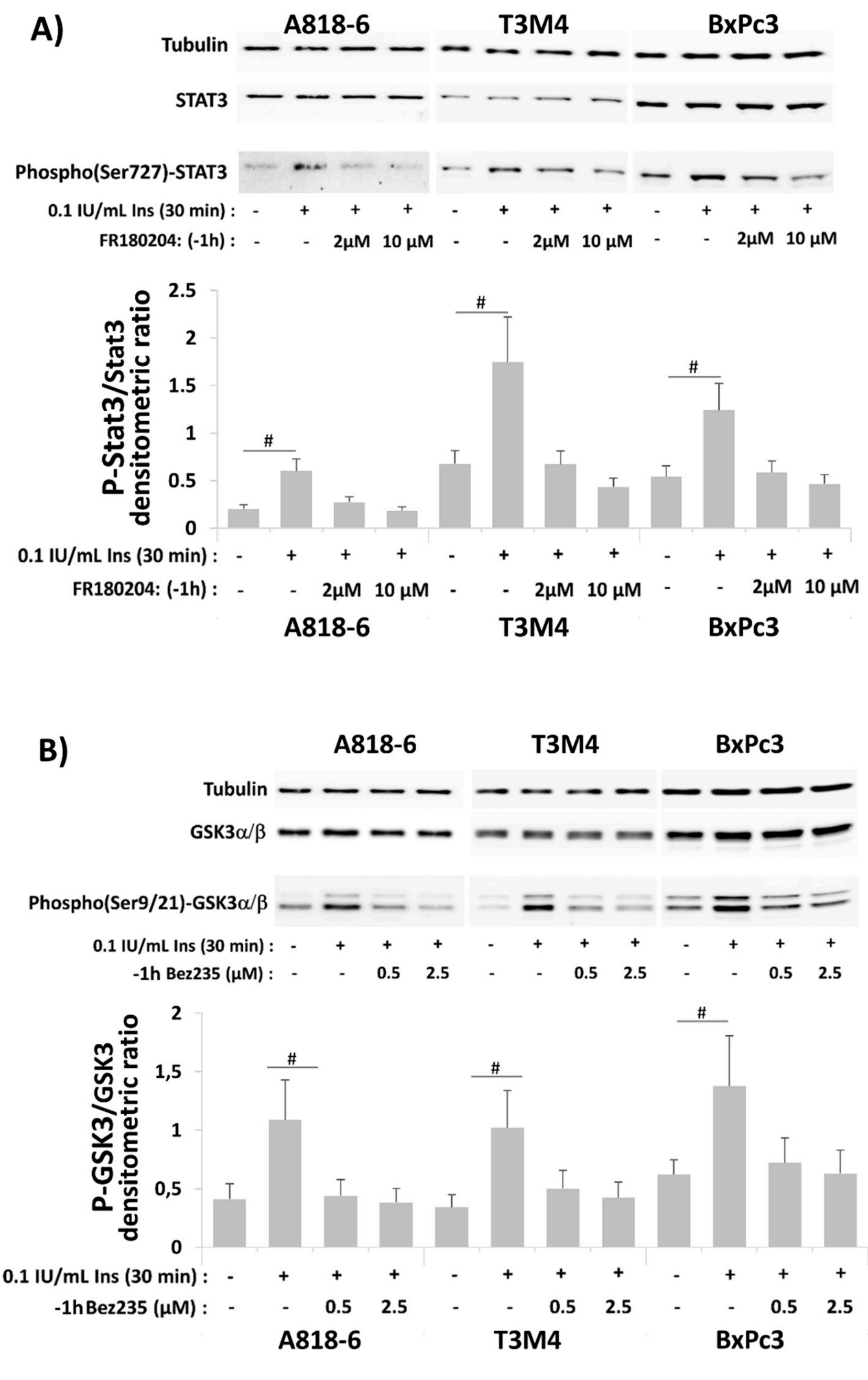

Figure S2. Validation of AKT inhibition by Bez235 and ERK1/2 inhibition by FR180204 in PDAC cells. Serum starved A818-6, T3M4 and BxPc3 cells were left untreated or were treated for 30 min with 0.1 IU/mL insulin either after pretreatment with **A)** the ERK inhibitor FR180204 (at 2 µM or 10 µM) or **B)** the PI3K inhibitor Bez235 (at 0.5 µM or 2.5 µM) for 1 h, or without pretreatment. Total cell lysates were submitted to Western blot analysis (representative result from 2 independent experiments) using **A)** Phospho(Ser727)-STAT3, STAT3 and tubulin antibodies or **B)** Phospho (Ser21/9)GSK3α/β, GSK3α/β and tubulin antibodies Representative blots from 4 independent experiments and band densitometry analyses (lower panels, $^{\#}p<0.05$, $n = 4$) are shown.

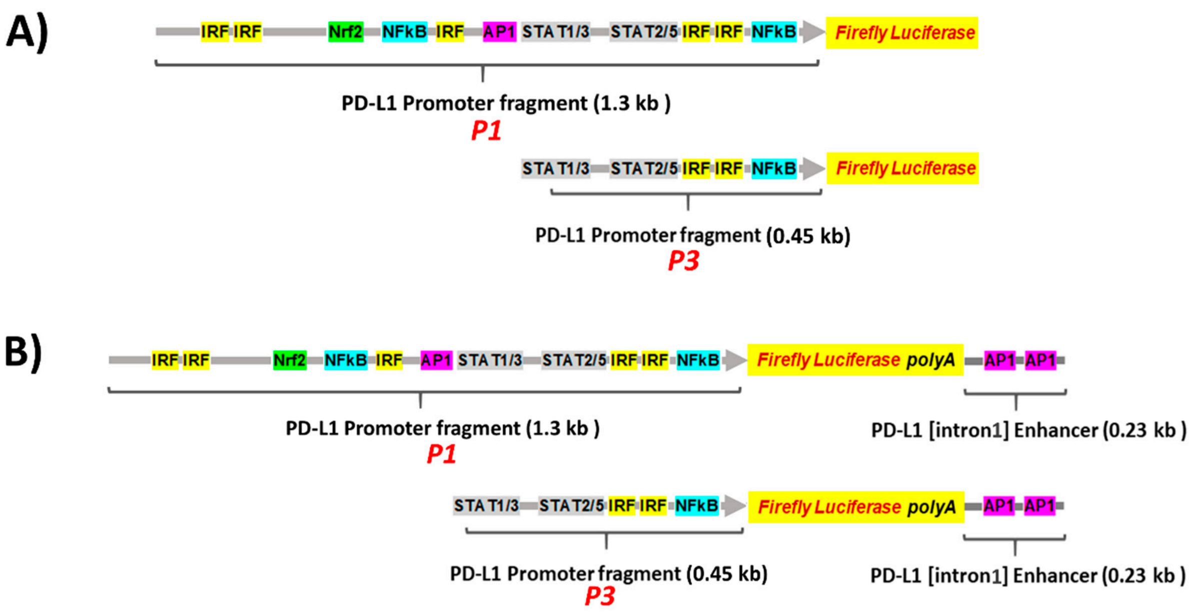

Figure S3. Structure of PD-L1 gene promoter constructs and the enhancer element from intron 1 (see also Figure 6 in the main body of the manuscript). **A),** Scheme of the 1.3 kb (P1) and 0.45 kb (P3) fragments from the human PD-L1 gene promoter cloned into the pGL3-luciferase vector. **B),** Scheme of the 1.3 kb (P1) and 0.45 kb (P3) fragments from the human PD-L1 gene promoter cloned into the pGL3-luciferase vector and insertion of the 0.23 kb enhancer elment from intron1 of the human PD-L1 gene at the 3′ end of the luciferase gene. The colored boxes highlight the established binding sites for the transcription factors IRF, STAT1/3, STAT2/5, Nrf2, NFκB and AP1.

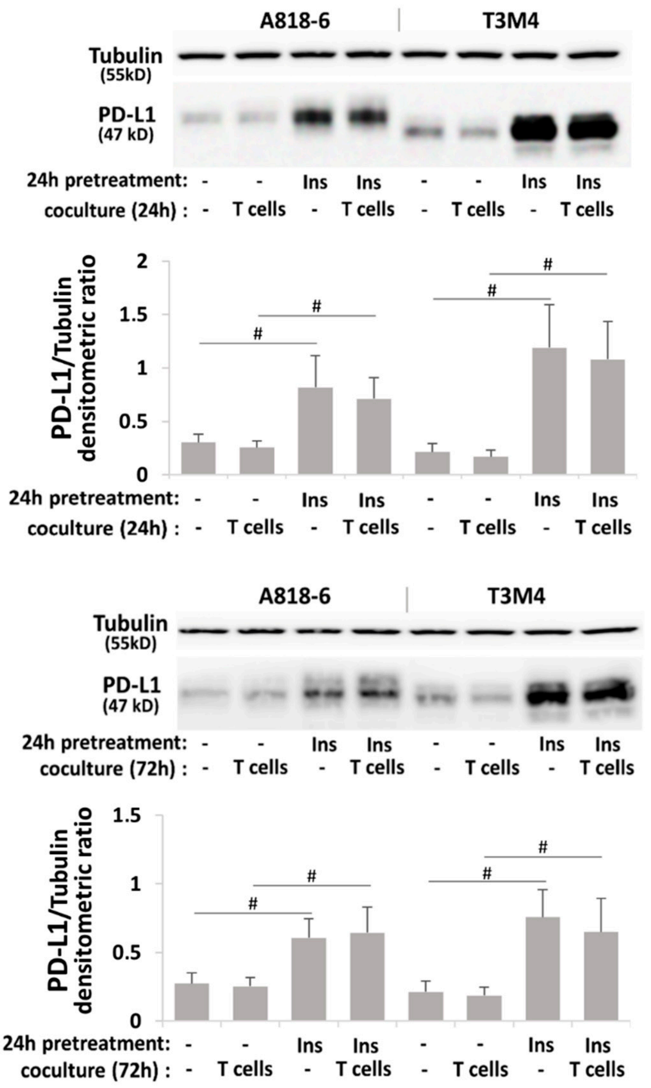

Figure S4. Insulin induced PD-L1 expression in A818-6 and T3M4 cell is maintained during T-Cell **cocultures.** Serum starved A818-6 and T3M4 cells (1x10^5) were left untreated (w/o) or treated with 0.1 IU/mL insulin (INS) for 24 h. Then, cells were co-cultured with 250,000 CFSE labelled and preactivated human CD8+ Tcells. At a final FCS concentration of 1 % (*v*/*v*), PDAC and T-cells were cocultured for 24h and 72 h. After removal of the T-cells and extensive washing, total cell lysates of the PDAC cells were submitted to Western blot analysis using PD-L1 and tubulin antibodies. Representative blots from 5 independent experiments and band densitometry analyses (lower panels, $^{\#}p < 0.05$, $n = 5$) are shown.

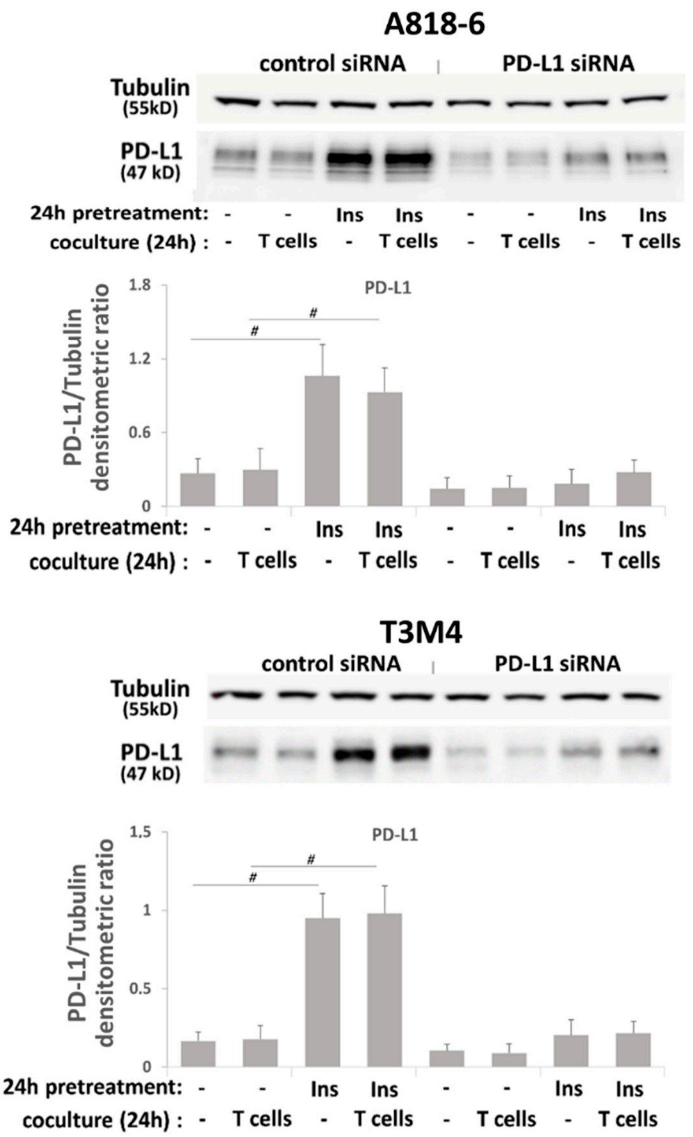

Figure S5. Knock down of PD-L1 expression in PDAC cells subject of subsequent T-cell coculture. A818-6 and T3M4 cells were pretreated with control or PD-L1 siRNA for 24 h, followed by serum starvation and subsequent treatment with 0.1 IE/mL insulin (Ins) or without (w/o) for 24 h. Then, cells were co-cultured with 250,000 CFSE labelled and preactivated human CD8+ Tcells. At a final FCS concentration of 1 % (*v*/*v*), PDAC and T-cells were cocultured for 24h. After removal of the T-cells and extensive washing, total cell lysates of the PDAC cells were submitted to Western blot analysis using PD-L1 and tubulin antibodies. Representative blots from 4 independent experiments and band densitometry analyses (lower panels, $^{\#}p < 0.05$, $n = 4$) are shown.
